# Consequences of the electronic tuning of latent ruthenium-based olefin metathesis catalysts on their reactivity

**DOI:** 10.3762/bjoc.11.158

**Published:** 2015-08-20

**Authors:** Karolina Żukowska, Eva Pump, Aleksandra E Pazio, Krzysztof Woźniak, Luigi Cavallo, Christian Slugovc

**Affiliations:** 1 Institute of Organic Chemistry, Polish Academy of Sciences, Kasprzaka 44/52, 01-224 Warszawa, Poland; 2 Institute of Chemistry and Technology of Materials, Graz University of Technology, NAWI Graz, Stremayrgasse 9, 8010 Graz, Austria; 3 Biological and Chemical Research Centre, Faculty of Chemistry, University of Warsaw, Żwirki i Wigury 101, 02-089; Warszawa, Poland; 4 Kaust Catalysis Center, Physical Sciences and Engineering Division, King Abdullah University of Science and Technology, Thuwal 23955-6900, Saudi Arabia

**Keywords:** DFT calculations, olefin metathesis, ring closing metathesis, ring-opening metathesis polymerisation, ruthenium

## Abstract

Two ruthenium olefin metathesis initiators featuring electronically modified quinoline-based chelating carbene ligands are introduced. Their reactivity in RCM and ROMP reactions was tested and the results were compared to those obtained with the parent unsubstituted compound. The studied complexes are very stable at high temperatures up to 140 °C. The placement of an electron-withdrawing functionality translates into an enhanced activity in RCM. While electronically modified precatalysts, which exist predominantly in the *trans*-dichloro configuration, gave mostly the RCM and a minor amount of the cycloisomerization product, the unmodified congener, which preferentially exists as its *cis*-dichloro isomer, shows a switched reactivity. The position of the equilibrium between the *cis*- and the *trans*-dichloro species was found to be the crucial factor governing the reactivity of the complexes.

## Introduction

Olefin metathesis is a catalytic process during which C–C double bonds are exchanged [1]. Since the first examples were published in the 1950s, many stunning accomplishments have been made in the field resulting in ever increasing interests in the method. Establishment of well-defined molybdenum- and ruthenium-based complexes lead to multitude of applications [2–4]. Especially, the latter class of compounds have gained attention due to their user-friendly character caused by a wide functional group-tolerance and high oxygen and moisture stability. Although a great progress has been made, the unsuitability of ruthenium complexes for high temperature applications remains one of the greatest challenges in the field.

**Figure 1:**
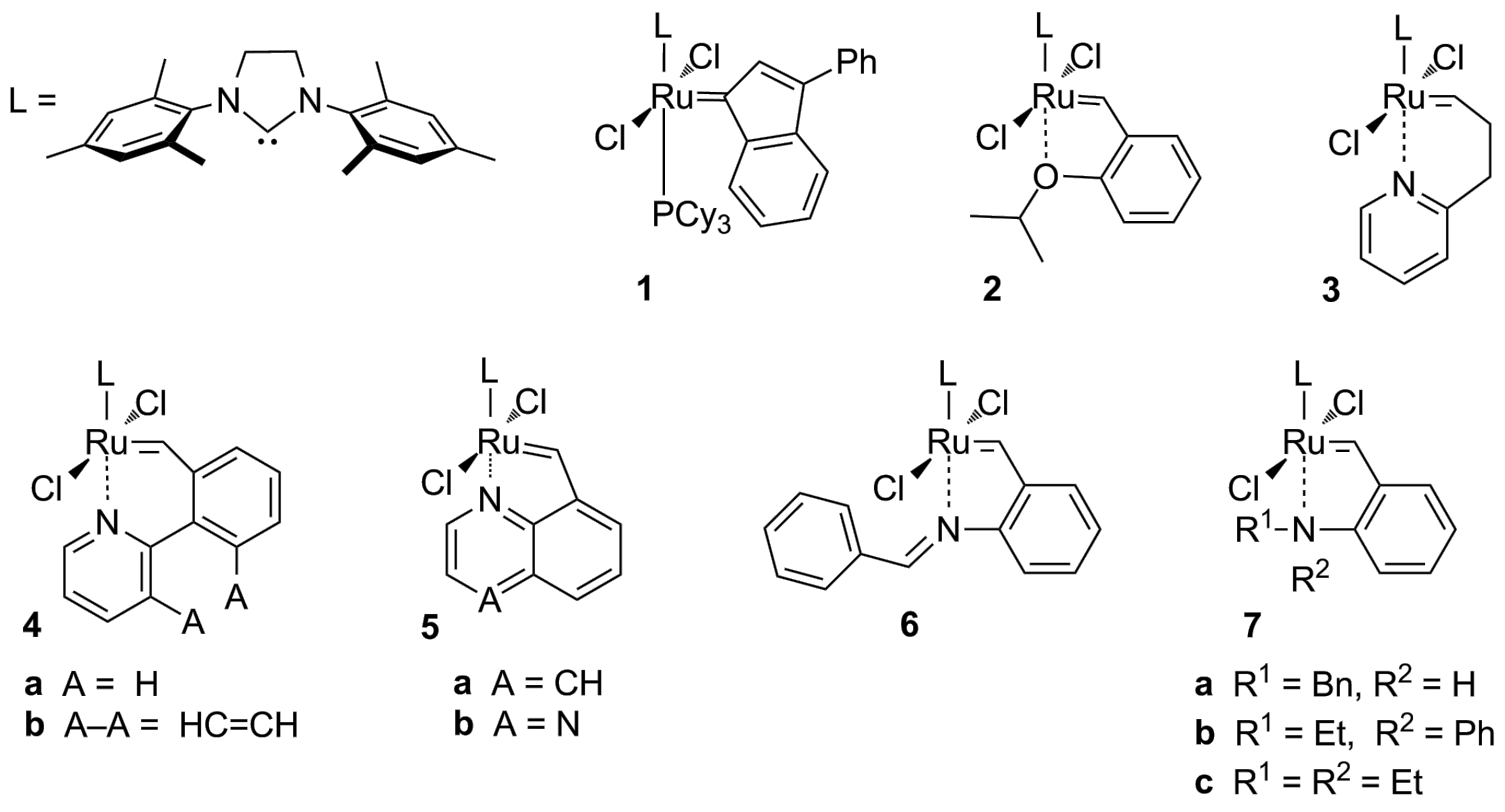
Selected ruthenium-based complexes.

Modifications in the basic structure of ruthenium-based olefin metathesis catalysts led to a diversification of catalytic profiles (Figure 1) [5–6]. Perhaps the most important one was the introduction of bidentate benzylidene ligands instead of simple alkylidenes, thus giving rise to the class of Hoveyda-type complexes with the parent compound **2** [7]. Further modifications of such systems followed. One of the most common is tuning of the properties of the benzylidene ligand so that modified reactivity of the resulting complex is achieved [8]. Various examples of such approaches have been published over the years. Particularly interesting results were obtained by substituting the oxygen atom with sulfur or nitrogen leading to a group of structurally diverse ruthenium chelates [9–11].

**Scheme 1:**
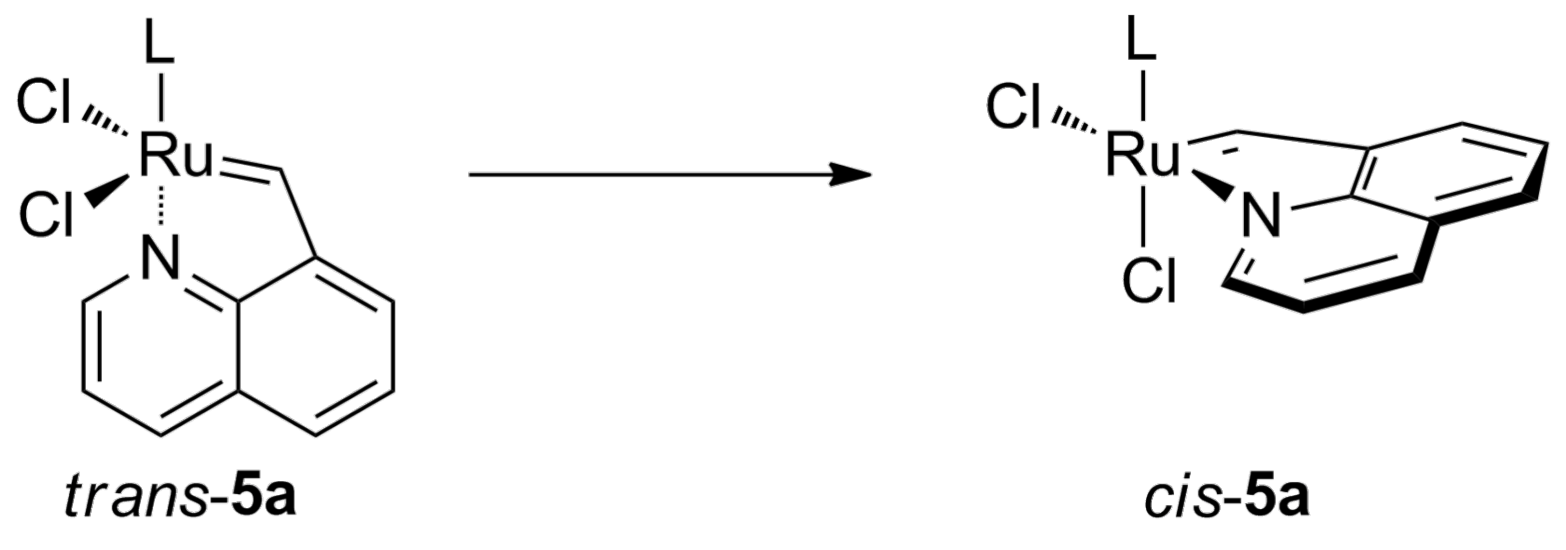
*trans–cis* Isomerization.

N-Based chelating complexes offer the advantage of a great availability of precursors for amine ligands making the number of possible structures virtually unlimited. Complexes incorporating alkylidene ligands based on aromatic [12–15] or aliphatic amines [16–17] and Schiff base patterns [18–22] have been prepared so far, exhibiting diverse activities ranging from very fast to very slow initiation. Furthermore, in those compounds, a *trans*–*cis* isomerization of the chloride ligands was observed in many cases (Scheme 1) [16]. This phenomenon has been widely discussed in literature with multiple reports of superior activity of *trans*-configured complexes. The general hypothesis is that the *trans*-dichloro form of the complex promotes metathesis whereas the *cis*-dichloro form is postulated to be metathetically inactive [23–24]. Thus, the *trans*–*cis* isomerization can be exploited for slowly releasing the olefin metathesis active species [25–26].

Aside from the interesting conformational behavior, nitrogen-chelated complexes possess some practical properties, namely they tend to be thermally stable, enabling applications at elevated temperatures. Being aware of the various advantages of such systems, we set out to study substituted quinolone–ruthenium chelates in view of their *trans*–*cis* susceptibility and its consequences.

## Results and Discussion

### Synthesis and characterization

**Scheme 2:**
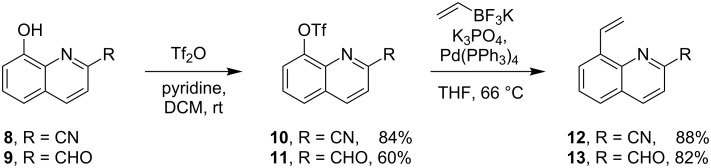
Synthesis of the ligand precursors.

The shortest pathway to obtain the desired ligands was chosen to provide access to starting materials. It was envisioned as a two-step sequence of triflation of commercially available substituted 8-hydroxyquinolines **8** and **9** followed by a Suzuki coupling as shown in Scheme 2 [14].

**Scheme 3:**
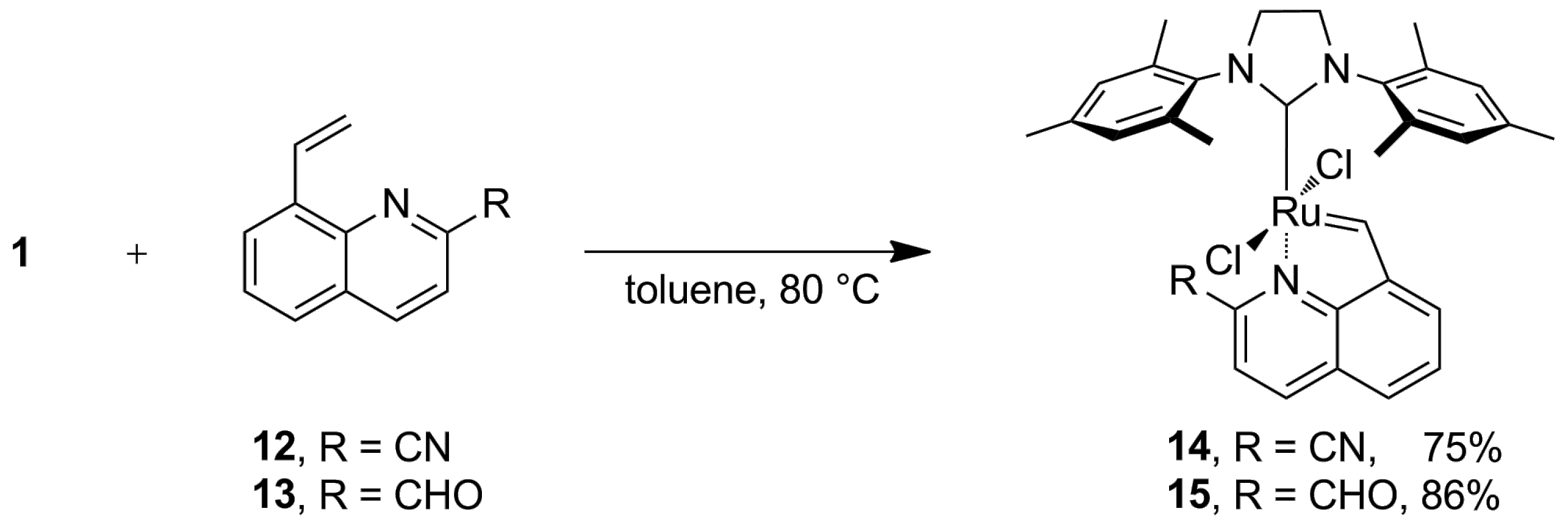
Synthesis of the ruthenium complexes.

The cyano-substituted compound **10** was obtained without difficulty, but esterification of compound **9** was problematic because of purification issues (cf. Supporting Information File 1) so an alternative pathway was established. Starting from 2-bromoaniline upon Doebner–Miller reaction and oxidation, we obtained the corresponding bromide derivative which was subsequently converted via Suzuki coupling into the carbene precursor **13**. Both compounds **12** and **13** were then used in a carbene exchange reaction with compound **1** conducted in toluene at 80 °C (see Scheme 3), releasing the desired complexes **14** and **15** as *trans*-dichloro isomers in good yields.

Based on the previously reported *trans*–*cis* isomerization of the parent quinoline-based complex **5a**, a potential isomerization of **14** and **15** was investigated. Reaction conditions for the isomerization of **5a** (complete isomerization after 6 days at 23 °C in CD_2_Cl_2_) [14] were proven to be ineffective for the isomerization of **14** and **15**, so that more forcing conditions were applied: Compounds **5a**, **14** or **15** were dissolved in CDCl_3_ and heated to 140 °C for 1 h in a microwave reactor.

When the reactions were performed in air, only decomposition of the complexes was observed. Upon using oxygen-free conditions a complete rearrangement of *trans*-**5a** to *cis*-**5a** in 30 min was obtained. Attempts, to prepare the *cis*-isomers of electronically modified complexes **14** and **15** in a similar fashion, however, turned out to be difficult. Using methods, such as starting from a pyridine containing the ruthenium complex, which is known to increase the *cis*-content, or increasing the exposure time of the catalyst up to 8 h in microwave at 140 °C in CDCl_3_ resulted in limited success.

The formyl-substituted complex **15** underwent isomerization, but only a mixture of 11% *cis*-**15** and 89% *trans*-**15** was obtained (cf. Supporting Information File 1). In the case of the CN-substituted compound **14**, on the other hand, no evidence for any isomerization could have been retrieved. These results suggest, that the introduction of the electron withdrawing substituent in 2-position changes the thermodynamic equilibrium favoring the *trans*- over the *cis*-isomers. In any case, a remarkable thermal stability of all studied compounds was found.

**Figure 2:**
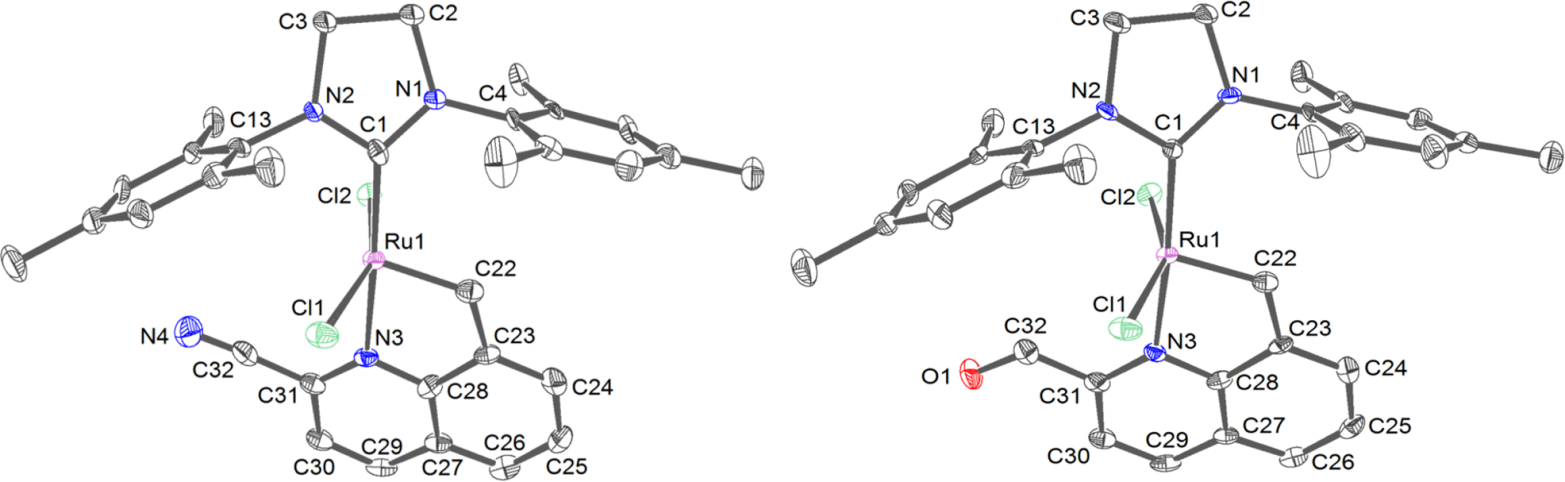
ADPs (atomic displacement parameters) and atoms labeling of the first molecule in the asymmetric part for **14** (left) and **15** (right). Thermal ellipsoids at 50% level of probability. Hydrogen atoms were omitted for clarity.

**Figure 3:**
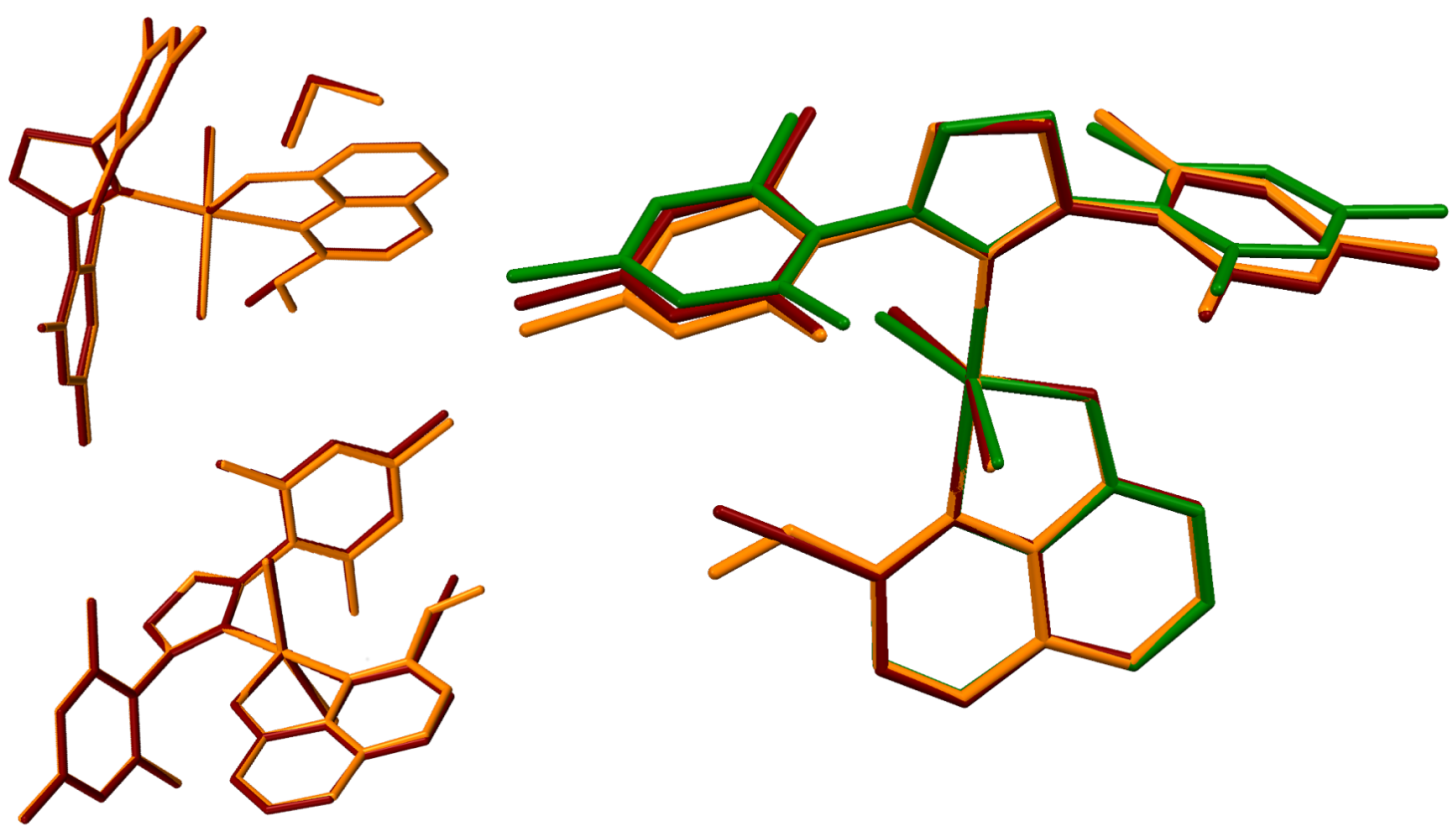
Superposition of the asymmetric parts of units’ cells in both investigated structures: an example of isostructural packing (left). Superposition (right side) *trans* forms of all studied molecules **5a** (green), **14** (ruby) and **15** (orange).

Intrigued by the observed phenomenon, we turned to structural studies. Structures of **14** and **15** have been determined using single-crystal X-ray diffraction. Each of them includes two molecules of the studied compound in the *trans*-conformation and one molecule of the solvent (dichloromethane) in the asymmetric part of the unit cell (Figure 2). Both crystals are isotypic, so the crystal packing is identical and lattice dimensions are very similar (Figure 3).

Bond lengths and angles of **5a**, **14** and **15** are very similar, so their different tendencies to form the corresponding *cis*-complexes could not be rationalized based on this dataset. The only feature worth mentioning is a slightly decreased Ru(1)-C(22) bond length in compound **15** compared to **5a** or **14** (1.829, 1.845 and 1.842 Å, respectively).

### DFT calculations

**Figure 4:**
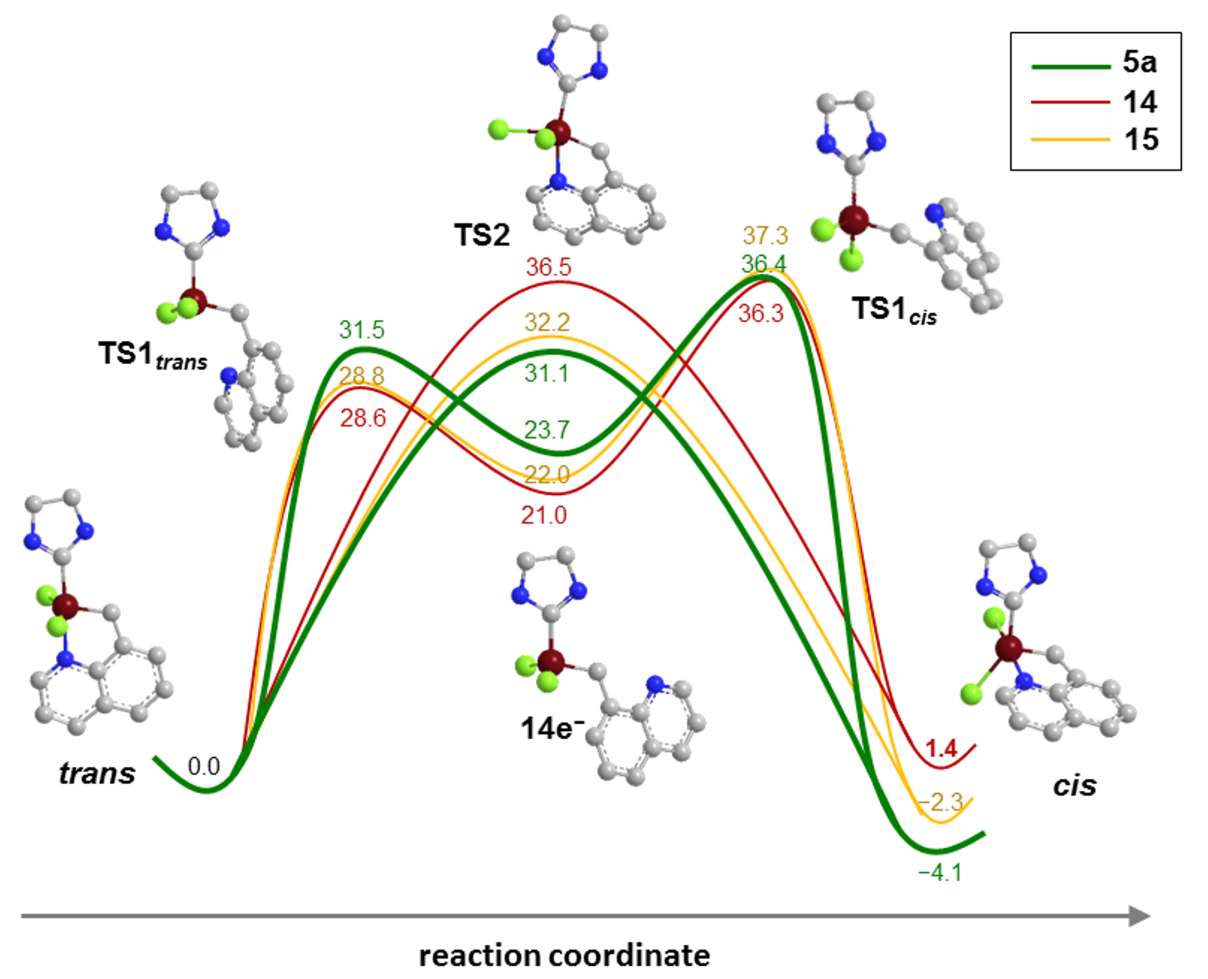
Energy profile of *trans–cis* isomerization, modelled in CH_2_Cl_2_, (Δ*E* in kcal/mol). Geometry optimizations BP86//SVP, solvent effects included by single point calculations, using M06//TZVP.

Density functional theory (DFT) calculations were performed with the aim to learn more about the compounds under investigation especially in view of their peculiar *trans*–*cis* isomerization behavior. Geometry optimizations were conducted by BP86//SVP, solvent effects were added by single point calculations using M06//TZVP functional (cf. Supporting Information File 1). The first question tackled concerned the anticipated coordination of the carbonyl group in compound **15** to form the corresponding 18-electron complex. Formation of the 18-electron compound is in principle feasible although it is endothermic by 2.6 kcal/mol. Thus, it can be concluded that preferably the corresponding 16-electron species is present in solution. Next, we investigated the relative stabilities of the *trans*-dichloro versus the *cis*-dichloro isomers. As already stated in literature, DFT calculations suggested a more preferential arrangement of **5a** in the *cis*-dichloro configuration [23–24]. Here, the equilibrium was investigated assuming solvation in CH_2_Cl_2_ and results revealed a preference for the *cis*-dichloro isomer in the case of **5a** (*cis*-**5a** is 4.1 kcal/mol more stable than *trans*-**5a**) and **15** (*cis*-**15** is 2.4 kcal/mol more stable than *trans*-**15**). In contrast, the cyano-group substituted compound **14** exists preferably in the *trans* configuration of the chloride ligands (*trans*-**14** is 1.4 kcal/mol more stable than *cis*-**14**). Because the *cis* isomer is better stabilized in solvents with high dielectric constants (such as CH_2_Cl_2_) than in solvents with low dielectric constants [27], the *trans*–*cis* energies were calculated in toluene (as an example for a solvent with low dielectric constant). In this case, **5a** is still preferably in the *cis* configuration (*cis*-**5a** is 1.3 kcal/mol more stable than *trans*-**5a**), while in **14** and **15** the *trans* configuration is favored (by 0.7 kcal/mol in case of **15** and 4.2 kcal/mol in case of **14**). Further, the energy profile for the isomerization reaction was investigated taking a dissociative and a concerted pathway into consideration (cf. Figure 4).

As disclosed earlier [23–24], the concerted pathway is the most likely operative for the isomerization of **5a**. The transition state **TS2** (which is the transition state for the concerted pathway) is 5.3 kcal lower in energy than the transition state for closing the chelating ligand towards the *cis*-dichloro configured form (**TS1*_cis_***). The energetic preference of the concerted over the dissociative pathway is decreasing when substituents in 2 position of the quinoline ligand are present. For **14** and **15** the transition state for the generation of the catalytically active 14-electron species (**TS1*_trans_***) is considerably lower compared to **5a**. These results go in hand with studies on electronically modified Hoveyda-type catalysts [28–29] and electronically modified ester-chelating benzylidene complexes [30]. Moreover, as already discussed, **TS2** becomes energetically more demanding so that for **14** and **15** the pathways for the isomerization (leading to the olefin metathesis inactive *cis*-dichloro form) becomes less important. Consequently, complex **15** and in particular **14** should be metathetically more active than their unsubsituted version **5a**.

### Activity in RCM

**Scheme 4:**
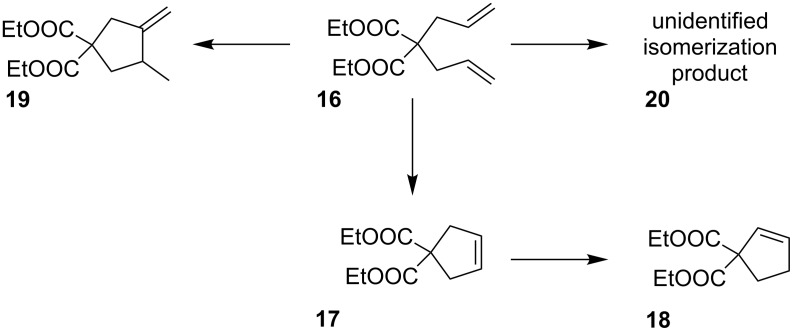
Possible reaction pathways of **16**.

The consecutive step of the research was devoted to exploring the activity of the obtained complexes in metathesis reactions. The preliminary choice was to conduct ring-closing metathesis (RCM) of diethyl diallylmalonate (**16**, Scheme 4).

**Figure 5:**
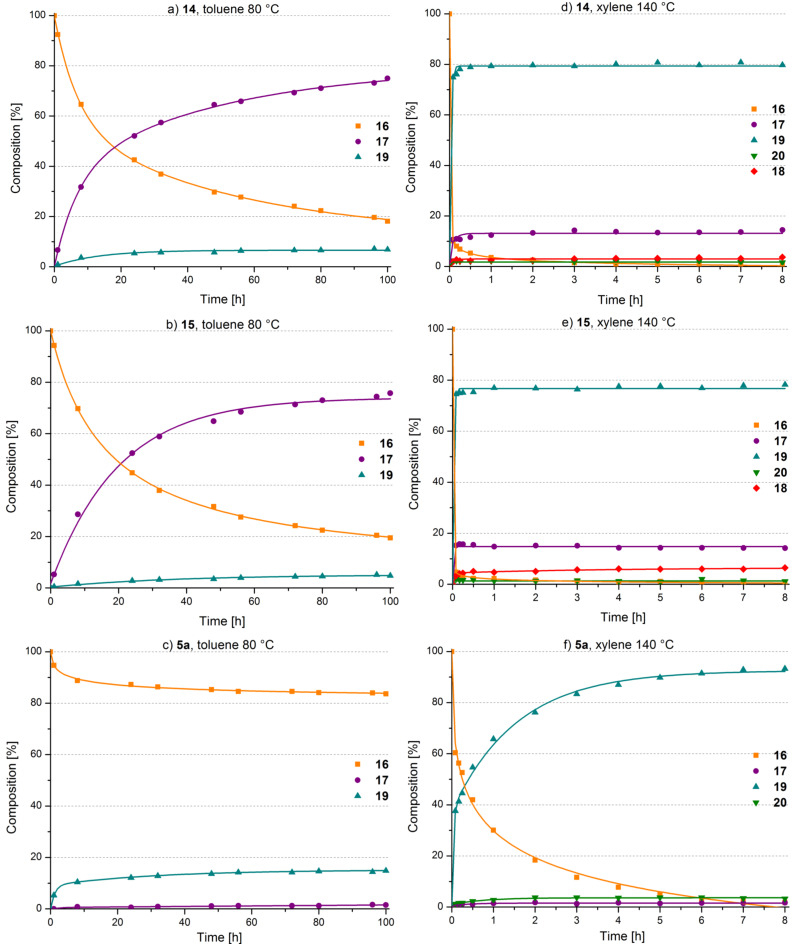
Time/conversion plots for the transformation of **16** catalyzed by 5 mol % of the *trans* isomers of *trans*-**5a**, **14** and **15**. Lines are intended as visual aids.

Initially examined conditions (DCM, rt) originate from the papers previously published by Grela’s group on the subject [14,31]. Unfortunately, along with F. Hoffmann-La Roche AG researchers [32] we were unable to fully reproduce the aforementioned results. The complex was inert at ambient conditions and only tests at elevated temperatures (80 or 140 °C) revealed catalytic activity of **5a**. When substrate **16** was subjected to RCM in high temperature conditions, a complex mixture of products was obtained (Figure 5). Upon GC and GC–MS analysis, structures of compounds **17**–**19** were determined (cf. Supporting Information File 1).

The substituted complexes were inert in ambient conditions similarly to the parent complex. The experiments at elevated temperature (toluene, 80 °C) revealed that precatalysts **14** and **15** led to higher conversions than the unsubstituted version **5a**. What is more interesting, both complexes exhibit prolonged activity and similar overall activity under the studied conditions. An interesting observation is that reactions catalyzed with **14** and **15** gave predominantly the RCM product (compound **17**) accompanied by a minor amount of a cycloisomerization-derived compound **19**. It is worth noting, that even after heating at 80 °C for 100 h the catalysts were still active, as can be assessed from the time/conversion plots (cf. Figure 5a and b). In contrast, the precatalyst **5a** promoted predominantly cycloisomerization albeit conversion was poor (cf. Figure 5c). Because all transformations were very slow at 80 °C the next series of test reactions was conducted at 140 °C in xylenes as the solvent (cf. Figure 5d–f). Electronically modified derivatives **14** and **15** promoted a faster transformation of **16**. In the first 10 min. about 90% of **16** was converted into about 80% cycloisomerization product **19** and about 12–16% RCM-product **17**, thus the selectivity changed upon raising the temperature. In addition, a minor amount of the isomerized RCM-product **18** and isomerized diethyl diallylmalonate **20** were detected in the reaction mixture. After about 1 h reaction time, the composition of the reaction mixture remained virtually unchanged upon prolonged heating.

**Figure 6:**
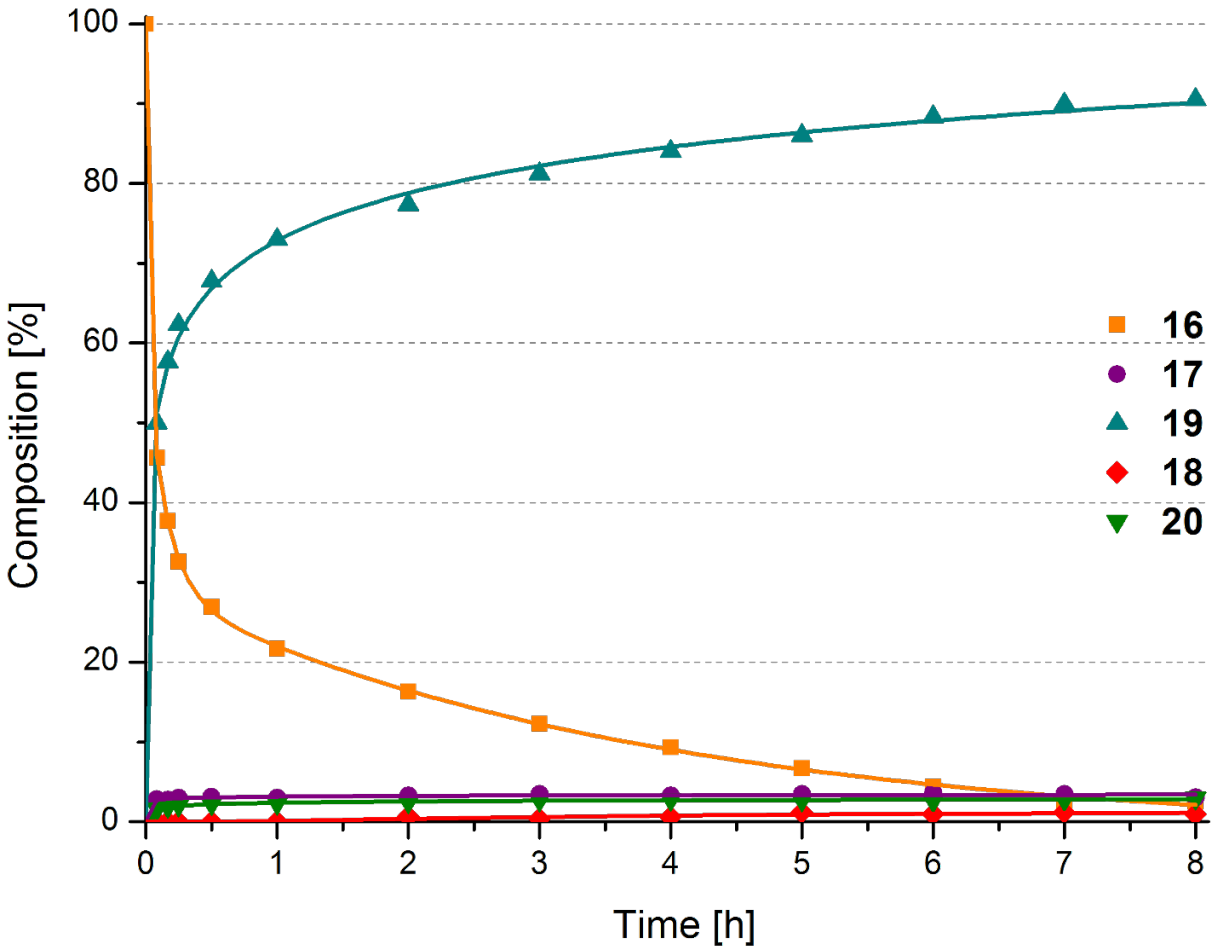
Composition of a reaction mixture after subjecting **16** to 5 mol % *cis*-**5a** in xylene, 140 °C. Lines are intended as visual aids.

At this high temperature, the precatalyst *trans*-**5a** resulted in a complete conversion of **16** which gave more than 90% cycloisomerization product **19** in 8 h. A small amount of isomerized diethyl diallylmalonate **20** and RCM-product **17** were also observed (cf. Figure 5f). Using *cis*-**5a** as the precatalyst resulted in similar results as can be seen in Figure 6. This is not especially peculiar as isomerization is conducted in the very same temperature range within less than 30 minutes meaning that the corresponding equilibrium is reached quickly.

Basing on the described experiments, it can be stated that precatalysts are thermally stable in the absence of oxygen and diethyl diallylmalonate at temperatures as high as 140 °C. Electronically modified precatalysts **14** and **15** initiate significantly faster than the parent precatalyst **5a** and when employed in RCM of diethyl diallylmalonate at 80 °C, those complexes gave predominantly the RCM product **17** accompanied with minor amounts of the cycloisomerization product **19**, while **5a** released predominantly the cycloisomerization product **19**.

Switching to 140 °C reaction temperature, all precatalysts released the cycloisomerization product **19** as the main product. These observations make again clear that the thermal stability of the precatalyst becomes irrelevant once it is in the presence of the substrate [33], because it is the thermal stability of the actual active species in the reaction mixture that governs the reaction outcome. In the present case, the methylidene complex formed during metathesis with the terminal olefin diethyl diallylmalonate is probably the most fragile species [32,34–36]. A recent work provides evidence that the catalytic species responsible for (cyclo)isomerization originates from decomposition of the methylidene [37]. Generally, the ability of olefin metathesis precatalysts to promote cycloisomerization [38] is known and has been widely researched both theoretically [39–40] and experimentally [41–43]. Moreover, the methylidene species alone are characterized by a certain degree of stability that is dramatically reduced in the presence of olefins, in particular, ethylene [38]. Accordingly, RCM reactions can be improved in terms of efficiency when ethylene is removed [44–46]. Based on these facts, the catalytic performance of the precatalysts **14** and **15** can be explained as follows: At 80 °C the actual active species is slowly released and performs mainly metathesis with diethyl diallylmalonate (**16**) leading, amongst other species, to the methylidene complex [47]. The latter is moderately stable under these conditions and participates in the catalytic RCM cycle. A concurring decomposition reaction of the methylidene or another Ru-species is responsible for the cycloisomerization side reaction. Further, the latter species is not able to isomerize the educt or the RCM product. Upon increasing the temperature to 140 °C, the said decomposition reaction is faster leading to the observed switch of reactivity in favor of the cycloisomerization pathway and isomerization of the educt as well as that of the product is observed. However, the results for **5a** as the precatalyst make clear that the thermal stability of the methylidene is not the only factor governing the outcome of the studied reactions. If the stability of the methylidene were the only crucial factor in all cases, the product distributions from reactions with **5a** would be similar to those from **14** and **15**. This is definitely not the case. Therefore, it can be assumed that in case of **5a** another, yet unknown decomposition reaction is responsible for the occurrence of the cycloisomerization reaction.

### Use as initiators in ROMP

**Figure 7:**
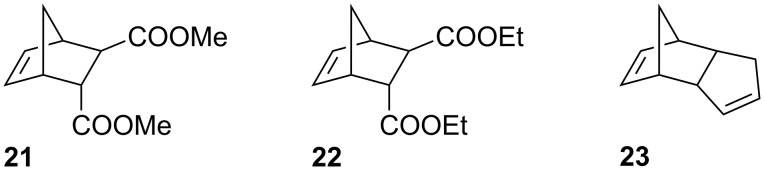
Monomers utilized in model ROMP reactions.

In the next step, compounds **5a**, **14** and **15** were tested as initiators in ring-opening metathesis polymerization (ROMP). The active species in ROMP (i.e., the propagating species) can be considered more stable than the methylidene intermediate in RCM, particularly when norbornenes such as *endo*,*exo*-bicyclo[2.2.1]hept-5-ene-2,3-dicarboxylic acid dimethyl ester (**21**) are polymerized (Figure 7) [48].

First, 300 equivalents of monomer **21** were polymerized with 1 equivalent initiator (**5a**, **14** or **15**) at 110 °C in toluene ([**21**] = 0.1 M) for 2 days. Initiator **5a** polymerized 90% of **21** and the corresponding polymer was characterized by a number average molecular weight (*M*_n_) of 557.0 kg·mol^−1^ (polydispersity index, PDI = 1.9) as examined by size exclusion chromatography (SEC) in THF against poly(styrene) standards. Initiator **15** gave 93% monomer-conversion and the resulting polymer exhibited a *M*_n_ of 516.0 kg·mol^−1^ (PDI = 2.2) and **14** gave the highest conversion (98%) and the shortest polymer strands (*M*_n_ = 326.7 kg·mol^−1^; PDI = 2.2).

**Figure 8:**
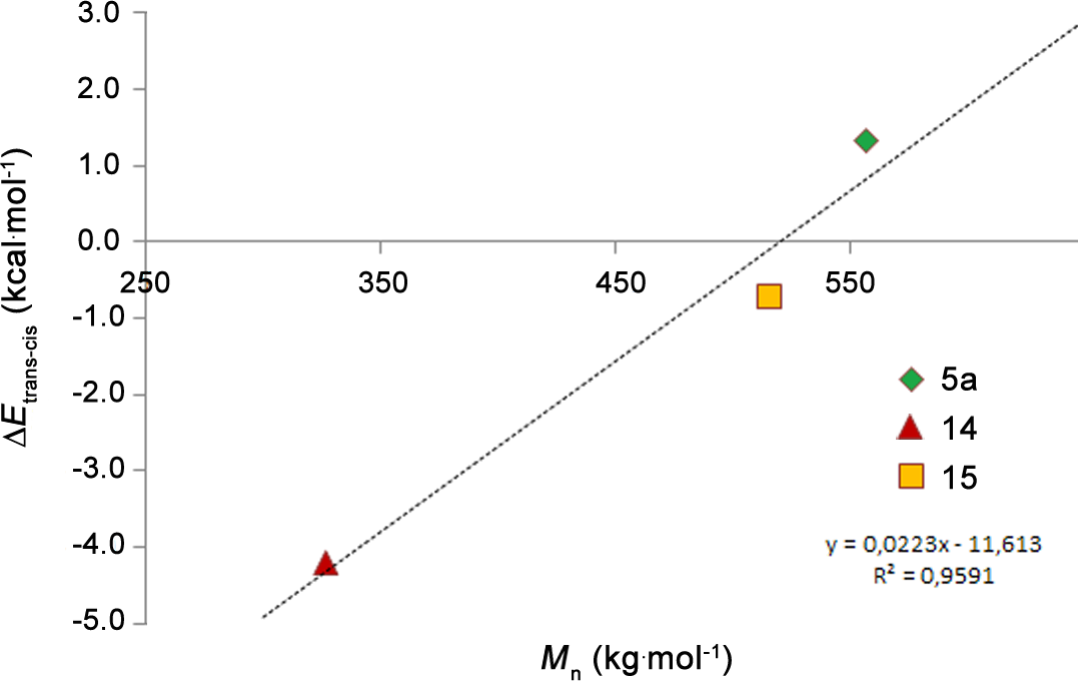
Number-average molecular weight (*M*_n_) of poly-**21** prepared with initiators **5a**, **14** and **15** plotted against the theoretically determined difference of *trans–cis* energies (solvation model PCM, solvent toluene).

The *M*_n_ values allow for an indirect relative assessment of the initiation efficacy [49–54], because they are proportional to the ratio of the propagation rate (*k*_p_) and the initiation rate constant (*k*_i_), provided that no secondary metathesis occurs. In this case, *M*_n_ is only dependent on *k*_i_, because in all cases the same propagating species occurs and *k*_p_ is the same. Accordingly, initiator **14** exhibits the highest initiation efficacy and initiator **5a** the lowest. Analyzing these data as disclosed previously, a linear correlation between the *M*_n_ values and the difference between the calculated thermodynamic stabilities of the *trans*- and the *cis*-dichloro configured isomers (Δ*E_trans_*_−_*_cis_*) can be established (Figure 8). This correlation suggests that the initiation efficacy is above all determined by the position of the *trans*–*cis* equilibrium which can be quickly reached at 110 °C [29,55].

**Figure 9:**
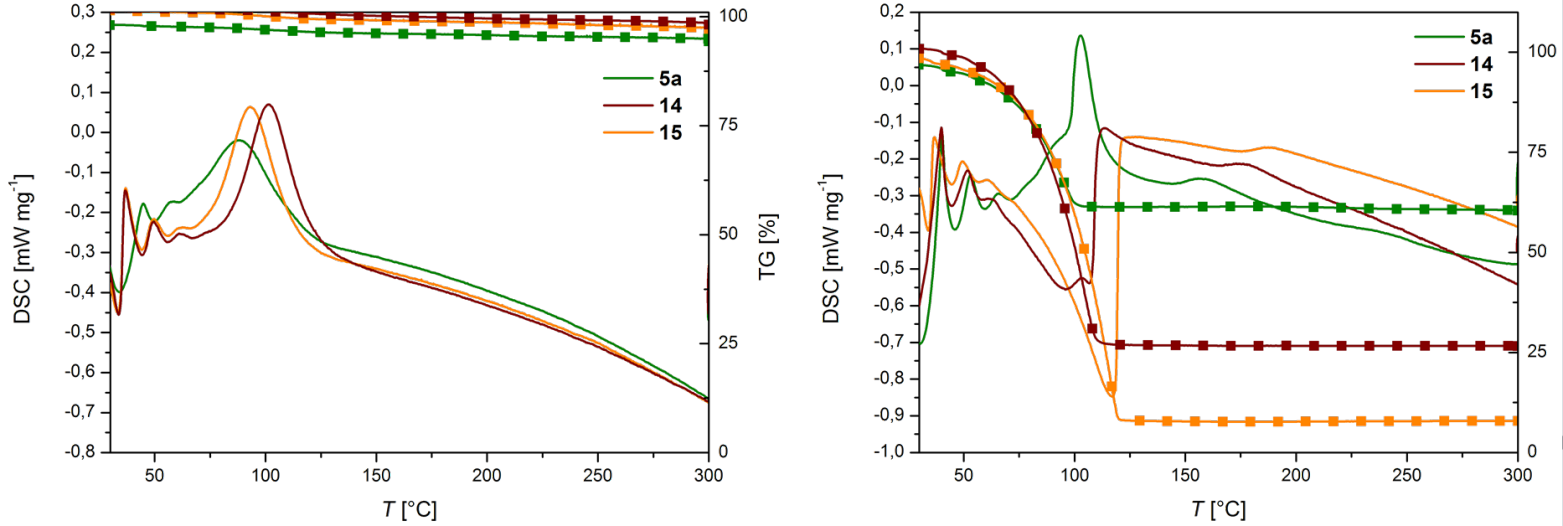
STA analysis of polymerization of **22** (left) and **23** (right), initiated by **5a**, **14** and **15**. Reaction conditions: [**22**]:[initiator] = 500 and [**23**]:[initiator] = 10000:1. Heating rate: 3 K/min. Big symbols: thermogravimetric analysis (TGA); no symbols: differential scanning calorimetry (DSC).

In the second step, the initiators were tested in neat monomer using simultaneous thermal analysis (STA) for monitoring the polymerizations. As the monomer, either *endo*,*exo*-bicyclo[2.2.1]hept-5-ene-2,3-dicarboxylic acid diethyl ester (**22**) or dicylcopentadiene (**23**) were used (cf. Figure 7). A distinctly changed initiation trend was observed under these reaction conditions. Initiator **5a** started the polymerization at the lowest temperature (onset of the polymerization exotherm at approx. 60 °C; cf. Figure 9, left) while the highest latency was found for initiator **14** (onset at approx. 75 °C). While initiators **14** and **15** exhibited rather sharp exotherms for the polymerization of monomer **22**, a much broader shape was found in the case of **5a**. This shape can be explained by assuming, that the isomerization of *trans*-**5a** to *cis*-**5a** is a concurring reaction, thus slowing down the polymerization reaction. The reason for the unexpectedly, at first sight, delayed initiation of **14** and **15** can most probably be attributed to steric effects. It is known that a coordination at the free coordination site (*trans* to the carbene ligand) can activate the (pre-)catalyst by lowering the energy barrier **TS1*_trans_*** to reach the active 14-electron species [14,56–57]. As both substituents, the CHO and the CN group, sterically shield the vacant coordination site, it is easily conceivable that such substrate-induced activation is impeded.

This effect turned out to be of particular relevance when the polymerization of dicyclopentadiene is regarded. Because polymerizations were carried out in open reaction vessels, the retro-Diels–Alder reaction of **23**, releasing volatile cyclopentadiene, is a concurring reaction and responsible for the low(er) polymer yields and pronounced endothermic signals in the DSC traces (cf. Figure 9 right) [58]. While initiator **5a** shows an appealing performance in polymerizing **23**, the electronically modified congeners **14** and **15** are not particularly interesting for this application.

## Conclusion

The present work introduced two ruthenium-based olefin metathesis catalysts/initiators featuring electronically modified quinoline-based chelating carbene ligands. Their reactivity in RCM and ROMP reactions was tested and results were set in comparison to those obtained with the parent compound, bearing the unsubstituted quinoline-based chelating carbene. The entire set of compounds is very stable at high temperatures up to 140 °C in the absence of oxygen and metathesis substrates. Electronic modification of the quinoline moiety changes the position of the *trans*–*cis* equilibrium as shown experimentally and theoretically. At the same time, electronic modification lowers the transition state energy for the generation of the catalytically active 14-electron species and increases the energy barrier for the transformation into the corresponding *cis*-dichloro isomers. Both effects translate into an enhanced activity in RCM at 80 °C when compared to the unmodified catalyst. In particular, the position of the *trans*–*cis* equilibrium is the most crucial factor governing the reactivity of the complexes. While electronically modified precatalysts which exist predominantly in *trans*-dichloro configuration gave mostly RCM and minor amounts of cycloisomerization product, the unmodified congener which preferentially exists in its *cis*-dichloro isomer, shows a switched reactivity. The reactivity switch is most probably caused by different substrate-induced decomposition reactions being responsible for the occurrence of the cycloisomerization reaction, which are more important at higher temperatures of 140 °C. In ROMP, again the position of the *trans*–*cis* equilibrium is the most crucial factor governing the initiation efficacy. Additionally, it has been shown, that steric effects of the substitution are responsible for an altered order of initiation behavior when polymerizations are conducted in bulk.

## Experimental

**Preparation of 14 and 15.** Precursor complex **1** (0.5 mmol, 475 mg) and the respective styrene derivative (0.55 mmol) were put in a Schlenk tube under argon. Reagents were dissolved in anhydrous toluene (25 mL) and the reaction was heated at 80 °C for about an hour. Then the solvent was evaporated and the mixture was purified by flash chromatography using eluents *c*-hexane/ethyl acetate 10:1 to 1:1 v/v. The solvent was evaporated. The product was then re-dissolved in dichloromethane and cold *n*-heptane was added to yield the product **14** as dark brown crystals (0.37 mmol, 242 mg, 75%). ^1^H NMR (CD_2_Cl_2_) δ 2.41 (s, 6H), 2.49 (s, 12H), 4.16 (s, 4H), 7.07 (s, 4H), 7.48–7.53 (m, 2H), 7.72 (dd, *J* = 0.9, 7.2 Hz, 1H), 8.24 (dd, *J* = 0.9, 8.3 Hz, 1H), 8.33 (d, *J* = 8.5 Hz, 1H), 16.95 (s, 1H) ppm; ^13^C NMR (CD_2_Cl_2_) δ 19.0, 20.9, 51.8, 116.1, 117.5, 122.6, 128.1, 129.4, 129.7, 133.9, 134.0, 134.6, 136.2, 138.3, 138.9, 146.8, 155.6, 209.4, 285.5 ppm; IR (KBr) ν: 3320, 3042, 3004, 2949, 2912, 2855, 2837, 2810, 2237, 1959, 1704, 1682, 1601, 1586, 1556, 1478, 1454, 1445, 1427, 1418, 1398, 1378, 1326, 1315, 1289, 1256, 1217, 1199, 1176, 1148, 1133, 1102, 1061, 1036, 1014, 985, 966, 930, 911, 879, 848, 822, 813, 792, 777, 752, 734, 721, 701, 653, 643, 622, 580, 533, 498, 428, 415 cm^−1^; HRMS (ESI) (*m*/*z*): [M] calcd, 644.1048; found, 644.1041.

Complex **15** was prepared analogously yielding dark brown crystals (0.43 mmol, 280 mg, 86%). ^1^H NMR (CD_2_Cl_2_) δ 2.43–2.55 (m, 16H), 4.19 (s, 4H), 7.11 (s, 4H), 7.48–7.55 (m, 1H), 7.72 (dd, *J* = 0.9, 7.2 Hz, 1H), 7.78 (d, *J* = 8.5 Hz, 1H), 8.28 (dd, *J* = 0.9, 8.2 Hz, 1H), 8.35 (d, *J* = 8.3 Hz, 1H), 8.96 (d, *J* = 0.6, Hz, 1H), 17.11 (s, 1H) ppm; ^13^C NMR (CD_2_Cl_2_) δ 19.1, 20.9, 51.6, 117.0, 122.8, 129.2, 130.8, 133.4, 134.3, 136.0, 138.9, 139.0, 145.9, 152.2, 156.3, 190.0, 210.6, 288.2 ppm; IR (KBr) ν: 3003, 2952, 2912, 2854, 2734, 2232, 1950, 1734, 1694, 1605, 1584, 1551, 1480, 1419, 1401, 1379, 1319, 1294, 1261, 1222, 1174, 1154, 1138, 1092, 1036, 987, 929, 910, 887, 846, 813, 794, 775, 732, 699, 680, 644, 591, 578, 535, 419 cm^−1^; HRMS (ESI) (*m*/*z*): [M − 2Cl + H]^+^ calcd, 578.1745; found, 578.1732.

## Supporting Information

File 1 Full experimental section along with all the synthetic procedures and analytical data of the obtained compounds.
