# Supplementary material for: Consequences of the electronic tuning of latent ruthenium-based olefin metathesis catalysts on their reactivity
Source: Beilstein J Org Chem. 2015 Aug 20;11:1458–68. doi: 10.3762/bjoc.11.158 (PMC4578431; doi:10.3762/bjoc.11.158)
Supplement: File 1 — Full experimental section along with all the synthetic procedures and analytical data of the obtained compounds. [file Beilstein_J_Org_Chem-11-1458-s001.pdf]

Supporting Information
for

Consequences of the electronic tuning of latent ruthenium-based olefin metathesis catalysts on their reactivity

Karolina Żukowska[1,*] , Eva Pump[2], Aleksandra E. Pazio[3], Krzysztof Woźniak[3], Luigi Cavallo[4], Christian Slugovc[2,*]

Address: [1]Institute of Organic Chemistry, Polish Academy of Sciences, Kasprzaka 44/52, 01-224 Warszawa, Poland; [2]Institute of Chemistry and Technology of Materials, Graz University of Technology, NAWI Graz, Stremayrgasse 9, 8010 Graz, Austria, [3]Biological and Chemical Research Centre, Faculty of Chemistry, University of Warsaw, Żwirki i Wigury 101, 02-089; Warszawa, Poland and [4]Kaust Catalysis Center, Physical Sciences and Engineering Division, King Abdullah University of Science and Technology, Thuwal 23955-6900, Saudi Arabia

Email: Karolina Żukowska - karolina.zukowska@gmail.com; Christian Slugovc - slugovc@tugraz.at
*Corresponding author

Full experimental section along with all the synthetic procedures and analytical data of the obtained compounds

Content

1. General information ... s2
2. Preparative experiments ... s2
   2.1. Esterification with triflic anhydride ... s2
   2.2. Suzuki coupling ... s3
   2.3. Alternative synthesis of **13** ... s4
   2.4. Synthesis of ruthenium complexes ... s5
   2.5. NMR spectra of compounds **10**–**15** ... s6
   2.6. General procedure of ring-closing metathesis reactions ... s12
   2.7. GC MS analyses ... s13
   2.8. Simultaneous Thermal Analysis (STA) ... s16
      2.8.1. Sample preparation for STA measurements with DCPD (**23**) ... s16
      2.8.2. Sample preparation for STA measurements with diethylester-norbornene (**22**) ... s16
      2.8.3. Polymerization of dimethylester-norbornene (**21**) in solution ... s16

2.9. *trans–cis* Isomerization s17
2.9.1. NMR studies s17
2.9.2. Preparation of *cis*-**5a** s18
2.9.3. Preparation of *cis*-**15** s19
3. X-ray crystallographic details s20
4. Details of computational approach s23

1. General information

All reactions were carried out under Ar in pre-dried glassware using Schlenk techniques. Toluene and tetrahydrofurane were dried by standard methods; other solvents were used as obtained. Flash column chromatography was performed using silica gel 60 (230–400 mesh). ^{1}H and ^{13}C NMR spectra were recorded on Agilent 400 MHz or Bruker Avance 300 MHz spectrometers, unless noted otherwise the 400 MHz was used. Chemical shifts (δ) are given in ppm relative to the solvent signals ($CDCl_3$, CD_2Cl_2), coupling constants (*J*) are given in Hz. Wavenumbers in the IR spectra are given in cm^{-1}. Gel permeation chromatography (GPC) was used to determine molecular weights and the polydispersity index. Measurements were carried out in THF with the following arrangement: a Merck Hitachi L6000 pump, separation columns of Polymer Standards Service (5 µm grade size), and a refractive-index detector from Wyatt Technology. For calibration polystyrene standards purchased from Polymer Standard Service were used. Simultaneous thermal analysis (STA) measurements were performed with a Netzsch Simultaneous Thermal Analyzer STA 449C (aluminum crucibles from Netzsch) operated with a helium flow rate of 50 mL/min in combination with a protetctive flow of 8 mL/min. GC–MS measurements were run on a Perkin Elmer Clarus 680 SQ8C with InterCap SMS/Sil column. IR spectra, MS and elemental analyses were provided by Institute of Organic Chemistry, PAS, Warsaw.

2. Preparative experiments

2.1. Esterification with triflic anhydride

The respective alcohol (5.9 mmol) was put in a Schlenk tube under Ar and dissolved in anhydrous pyridine (2.4 mL). The solution was cooled down to 0 °C and a solution of triflic anhydride (7.1 mmol, 1.2 mL) in anhydrous DCM (7.2 mL) was added dropwise. The reaction was allowed to warm to rt, and was stirred overnight. Then water was added to the solution and the aqueous layer was extracted with dichloromethane (3 × 10 mL). The combined organic layers were dried and concentrated in vacuo. The crude product was purified by flash chromatography using eluents: *c*-hexane/ethyl acetate 10:1 to 1:1 v/v to provide the product.

10, white crystals (4.95 mmol, 1.49g, 84%). ^{1}H NMR ($CDCl_3$): 7.80-7.73 (m, 2H), 7.84 (d, *J* = 8.5 Hz, 1H), 7.96 (dd, *J* = 2.4, 7.2 Hz, 1H), 8.42 (d, *J* = 8.5 Hz, 1H) ppm. ^{13}C NMR ($CDCl_3$): 116.9, 117.4, 123.4, 125.1, 128.3, 129.2, 130.0, 134.9, 137.8, 141.1, 145.7 ppm. IR (KBr): ν 3103, 3055, 3032, 2237, 1984, 1846, 1780, 1629, 1585, 1561, 1498, 1462, 1433, 1419, 1403, 1383, 1313, 1252, 1240, 1214, 1204,

1165, 1143, 1133, 1068, 1046, 915, 885, 854, 821, 809, 774, 766, 713, 691, 636, 599, 577, 564, 550, 526, 498, 454, 424 cm^{-1}. MS (EI; m/z): 63 (21), 69 (29), 114 (45), 115 (19), 141 (100), 142 (22), 169 (36), 210 (25), 238 (62), 302 (44, $M^{+\bullet}$). Anal. Calcd for $C_{11}H_5F_3N_2O_3S$ (302.23): C, 43.71; H, 1.67; N, 9.27; S, 10.61. Found: C, 43.75; H, 1.57; N, 9.25; S, 10.65.

11, white crystals (3.54 mmol, 1.08g , 60%). ^1H NMR ($CDCl_3$): 7.73 (d, *J* = 1.6 Hz, 1H), 7.74 (s, 1H), 7.93-8.00 (m, 1H), 8.16 (d, *J* = 8.5 Hz, 1H), 8.41 (d, *J* = 8.5 Hz, 1H), δ 10.26 (s, 1H) ppm. ^{13}C NMR ($CDCl_3$): 118.9, 122.4, 128.3, 128.8, 131.4, 137.7, 193.1 ppm. IR (KBr): ν 3422, 3106, 3076, 2875, 1967, 1945, 1888, 1831, 1721, 1682, 1626, 1594, 1565, 1501, 1466, 1425, 1405, 1313, 1250, 1209, 1156, 1139, 1084, 1064, 1041, 995, 983, 974, 913, 882, 847, 824, 805, 763, 709, 667, 623, 595, 571, 542, 533, 509, 494, 467, 418, 411 cm^{-1}. MS (EI; m/z): 63 (30), 69 (32), 89 (57), 115 (29), 116 (95), 127 (62), 144 (50), 172 (70), 193 (25), 305 (100, $M^{+\bullet}$). Anal. Calcd for $C_{11}H_6F_3NO_4S$ (305.23): C, 43.28; H, 1.98; F, 18.67; N, 4.59; O, 20.97; S, 10.51. Found C, 43.28; H, 1.85; N, 4.43; S, 10.70.

2.2. Suzuki coupling

Tetrakis(triphenylphosphine)palladium (0.1 mmol, 116 mg), potassium vinyltrifluoroborate (1.25 mmol, 167 mg), tripotassium phosphate (3 mmol, 637 mg) and the respective triflate (1 mmol) were placed in a Schlenk tube under argon. Anhydrous THF (18 mL) was added and the solution was refluxed overnight under argon atmosphere. After this time the reaction mixture was cooled, water (10 mL) was added and the aqueous layer was extracted with dichloromethane (3 × 10 mL). The combined organic layers were dried and concentrated in vacuo. The crude product was purified by flash chromatography using eluents: *c*-hexane/ethyl acetate 10:1 to 1:1 v/v to provide the product.

12, white crystals (0.88 mmol, 158 mg, 88%). ^1H NMR ($CDCl_3$): 5.58 (dd, *J* = 1.3, 11.1 Hz, 1H), 6.05 (dd, *J* = 1.3, 17.8 Hz, 1H), 7.75- 7.65 (m, 2H), 7.81 (dd, *J* = 1.4, 8.2 Hz, 1H), 7.97 (dd, *J* = 11.1, 17.8 Hz, 1H), 8.08-8.02 (m, 1H), 8.28 (d, *J* = 8.4 Hz, 1H) ppm.^{13}C NMR ($CDCl_3$): 117.5, 117.9, 123.5, 127.0, 127.4, 129.0, 129.5, 132.0, 132.8, 137.1, 137.7, 145.8 ppm. IR (KBr): ν 3082, 3071, 2983, 2236, 1969, 1943, 1884, 1807, 1680, 1611, 1585, 1559, 1498, 1451, 1434, 1411, 1368, 1310, 1283, 1256, 1212, 1166, 1139, 1088, 1021, 987, 912, 894, 847, 813, 766, 714, 693, 665, 644, 603, 571, 554, 514, 439, 423 cm^{-1}. MS (EI; m/z): 179 (100), 180 (58, $M^{+\bullet}$). Anal. Calcd for $C_{12}H_8N_2$ (180.21): C, 79.98; H, 4.47; N, 15.55. Found: C, 79.60; H, 4.50; N, 15.43.

13, straw yellow crystals (0.82 mmol, 150mg, 82%). ^1H NMR ($CDCl_3$): 5.60 (dd, *J* = 1.3, 11.1 Hz, 1H), 6.09 (dd, *J* = 1.3, 17.8 Hz, 1H), 7.72-7.64 (m, 1H), 7.83 (dd, *J* = 1.2, 8.2Hz, 1H), 8.01-8.06 (m, 2H), 8.13 (dd, *J* = 11.1, 17.8 Hz, 1H), 8.30 (d, *J* = 8.4 Hz, 1H), 10.26 (s, 1H) ppm. ^{13}C NMR ($CDCl_3$): 117.1, 117.4, 126.4, 127.6, 129.3, 132.5, 137.4, 137.7, 194.1 ppm. IR (KBr): ν 3069, 2983, 2829, 2809, 1964, 1930, 1822, 1745, 1709, 1600, 1585, 1566, 1500, 1456, 1419, 1366, 1354, 1324, 1313, 1286, 1262, 1233, 1201, 1163, 1128, 1987, 1087, 1024, 1000, 905, 846, 813, 761, 711, 616, 593, 516, 468 cm^{-1}. MS (EI; m/z): 51 (9), 75 (9), 77 (14), 126 (12), 127 (16), 128 (10), 154 (64), 155 (17), 182 (100), 183 (99, $M^{+\bullet}$). Anal. Calcd for $C_{12}H_9NO$ (183.21): C, 78.67; H, 4.95; N, 7.65. Found: C, 78.71; H, 4.98; N, 7.51.

2.3. Alternative synthesis of 13

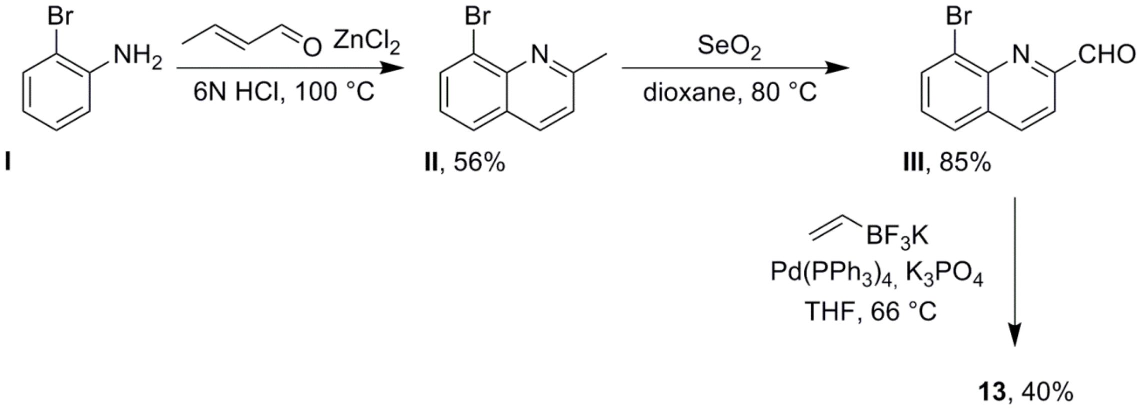

Scheme 1. Alternative synthesis of **13.**

According to a literature procedure[1] 2-bromoaniline (58.2 mmol, 10 g) was dissolved in 30 mL of 6 N HCl and stirred under reflux. Crotonaldehyde (64 mmol, 5.3 mL) was added dropwise. The reaction was refluxed overnight. It was then cooled down. 40 mL of diethyl ether were added followed by addition of anhydrous zinc chloride (58.2 mmol, 7.9 g). The reaction mixture was stirred for 15 minutes at room temperature, and further 15 minutes in 0 °C. The formed solid was filtered and washed with 2-propanol until the filtrate became colorless. Then it was washed with 40 mL of diethyl ether and dried. The solid was suspended in 30 mL of water and 10 mL of ammonium hydroxide were added. The mixture was vigorously shaken and extracted with diethyl ether. Combined organic phases were dried over magnesium sulfate and evaporated. The product was purified by CC to give **II,** as pale yellow solid with 56% yield (32.5 mmol, 7.2g). ^{1}H NMR ($CDCl_3$): 2.82 (s, 3H), 7.32 (t, *J* = 8.0 Hz, 2H), 7.73 (dd, *J* = 1.3, 8.1 Hz, 1H), 8.06-7.97 (m, 2H) ppm. ^{13}C NMR ($CDCl_3$): 25.9, 123.0, 124.3, 126.2, 127.6, 127.9, 133.2, 136.7, 145.0, 160.6 ppm. Analyses in accordance with literature reports.[1]

According to a literature procedure[2] selenium(IV) dioxide (41.5 mmol, 4.6 g) was dissolved in dioxane in a Schlenk tube under argon. The solution was heated to 80 °C. Then **II** (41.5 mmol, 9.2 g) was added. The reaction mixture was heated overnight, then cooled down to room temperature and filtered through neutral alumina. The solution was evaporated and the residue was washed with cold acetone to yield **III**, as a pale yelow solid with 85% yield (35.4 mmol, 8.3 g). ^{1}H NMR ($CDCl_3$): 7.51-7.59 (m, 1H), 7.89 (dd, *J* = 1.2, 8.2Hz, 1H), 8.10 (d, *J* = 8.4 Hz, 1H), 8.17 (dd, *J* = 1.3, 7.5 Hz, 1H), 8.34 (d, *J* = 8.4 Hz, 1H), 10.31 (d, *J* = 0.8 Hz, 1H) ppm. ^{13}C NMR ($CDCl_3$): 118.3, 126.3, 127.9, 129.7, 131.5, 134.4, 138.3, 145.2, 153.3, 193.6 ppm. Analyses in accordance with literature reports.[2]

Tetrakis(triphenylphosphine)palladium (1.77 mmol, 2 g), potassium vinyltrifluoroborate (44.2 mmol, 5.9 mg), tripotassium phosphate (106 mmol, 22.5 g) and compound **III** (35.4 mmol, 8.3 g) were placed in a Schlenk tube under argon. Anhydrous THF (250 mL) was added and the solution was refluxed overnight under argon atmosphere. After this time the reaction mixture was cooled, water (150 mL) was added and the aqueous layer was extracted with dichloromethane (3 × 100 mL). The combined organic layers were dried and concentrated in vacuo. The crude product was purified by

[1] Lin, N.; Yan, J.; Huang, Z.; Altier, C.; Li, M.; Carrasco, N.; Suyemoto, M.; Johnston, L.; Wang, S.; Wang, Q.; Fang, H.; Caton-Williams, J.; Wang. B., *Nucleic Acids Res.* **2007**, *35*, 1222–1229.
[2] Suess, D. L. M.; Peters, J. C., *Chem. Commun.* **2010**, *46*, 6554–6556.

flash chromatography using eluents: *c*-hexane/ethyl acetate 10:1 to 1:1 v/v to provide the product. Multiple column chromatographies and crystallizations resulted in **13** (14.3 mmol, 2.6 g) with 40% yield, spectroscopically pure as described part 2.2.

2.4. Synthesis of ruthenium complexes

Precursor complex **1** (0.5 mmol, 475 mg) and the respective styrene derivative (0.55 mmol) were put in a Schlenk tube under argon. Reagents were dissolved in anhydrous toluene (25 mL) and the reaction was heated in 80 °C for about an hour. Then the solvent was evaporated and the mixture was purified by a flash chromatography using eluents: *c*-hexane/ethyl acetate 10 : 1 to 1 : 1 v/v. The solvent was evaporated. It was then re-dissolved in DCM and cold *n*-heptane was added to produce crystals of the product. Crystals suitable for X-ray analysis were grown form the DCM / *n*-heptane mixture.

14, dark brown crystals (0.37 mmol, 242 mg, 75%). ^{1}H NMR (CD_2Cl_2): 2.41(s, 6H), 2.49 (s, 12H), 4.16 (s, 4H), 7.07 (s, 4H), 7.48-7.53 (m, 2H), 7.72 (dd, *J* = 0.9, 7.2 Hz, 1H), 8.24 (dd, *J* = 0.9, 8.3 Hz, 1H), 8.33 (d, *J* = 8.5 Hz, 1H), 16.95 (s, 1H) ppm. ^{13}C NMR (CD_2Cl_2): 19.0, 20.9, 51.8, 116.1, 117.5, 122.6, 128.1, 129.4, 129.7, 133.9, 134.0, 134.6, 136.2, 138.3, 138.9, 146.8, 155.6, 209.4, 285.5, ppm. IR (KBr): ν 3320, 3042, 3004, 2949, 2912, 2855, 2837, 2810, 2237, 1959, 1704, 1682, 1601, 1586, 1556, 1478, 1454, 1445, 1427, 1418, 1398, 1378, 1326, 1315, 1289, 1256, 1217, 1199, 1176, 1148, 1133, 1102, 1061, 1036, 1014, 985, 966, 930, 911, 879, 848, 822, 813, 792, 777, 752, 734, 721, 701, 653, 643, 622, 580, 533, 498, 428, 415 cm^{-1}. HRMS (ESI) (*m/z*): (M) Calcd 644.1048, found: 644.1041

15, dark brown crystals (0.43 mmol, 280 mg, 86%). ^{1}H NMR (CD_2Cl_2): 2.43-2.55 (m, 16H), 4.19 (s, 4H), 7.11 (s, 4H), 7.48-7.55 (m, 1H), 7.72 (dd, *J* = 0.9, 7.2 Hz, 1H), 7.78 (d, *J* = 8.5 Hz, 1H), 8.28 (dd, *J* = 0.9, 8.2 Hz, 1H), 8.35 (d, *J* = 8.3 Hz, 1H), 8.96 (d, *J* = 0.6, Hz, 1H), 17.11 (s, 1H) ppm. ^{13}C NMR (CD_2Cl_2): 19.1, 20.9, 51.6, 117.0, 122.8, 129.2, 130.8, 133.4, 134.3, 136.0, 138.9, 139.0, 145.9, 152.2, 156.3, 190.0, 210.6, 288.2 ppm. IR (KBr): ν 3003, 2952, 2912, 2854, 2734, 2232, 1950, 1734, 1694, 1605, 1584, 1551, 1480, 1419, 1401, 1379, 1319, 1294, 1261, 1222, 1174, 1154, 1138, 1092, 1036, 987, 929, 910, 887, 846, 813, 794, 775, 732, 699, 680, 644, 591, 578, 535, 419 cm^{-1}. HRMS (ESI) (*m/z*): [$(M – 2Cl + H)^+$] Calcd: 578.1745, found: 578.1732.

2.5. NMR spectra of compounds 10-15

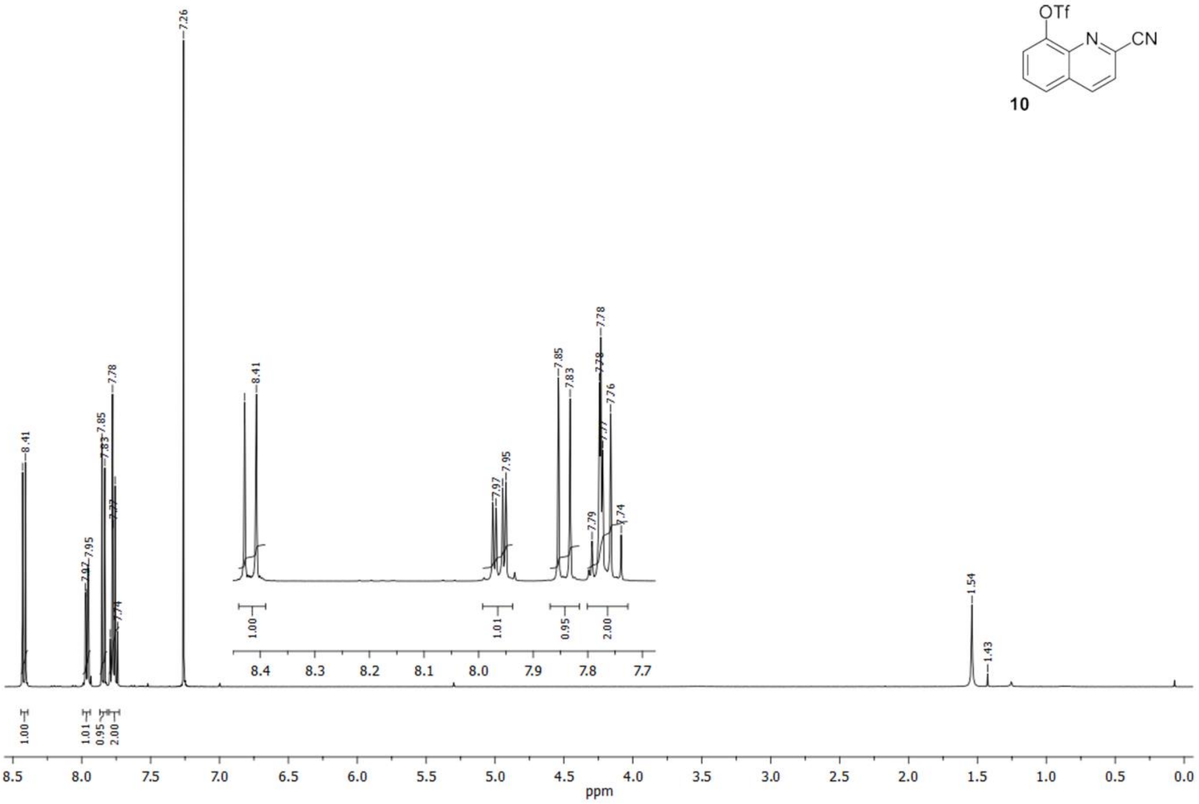

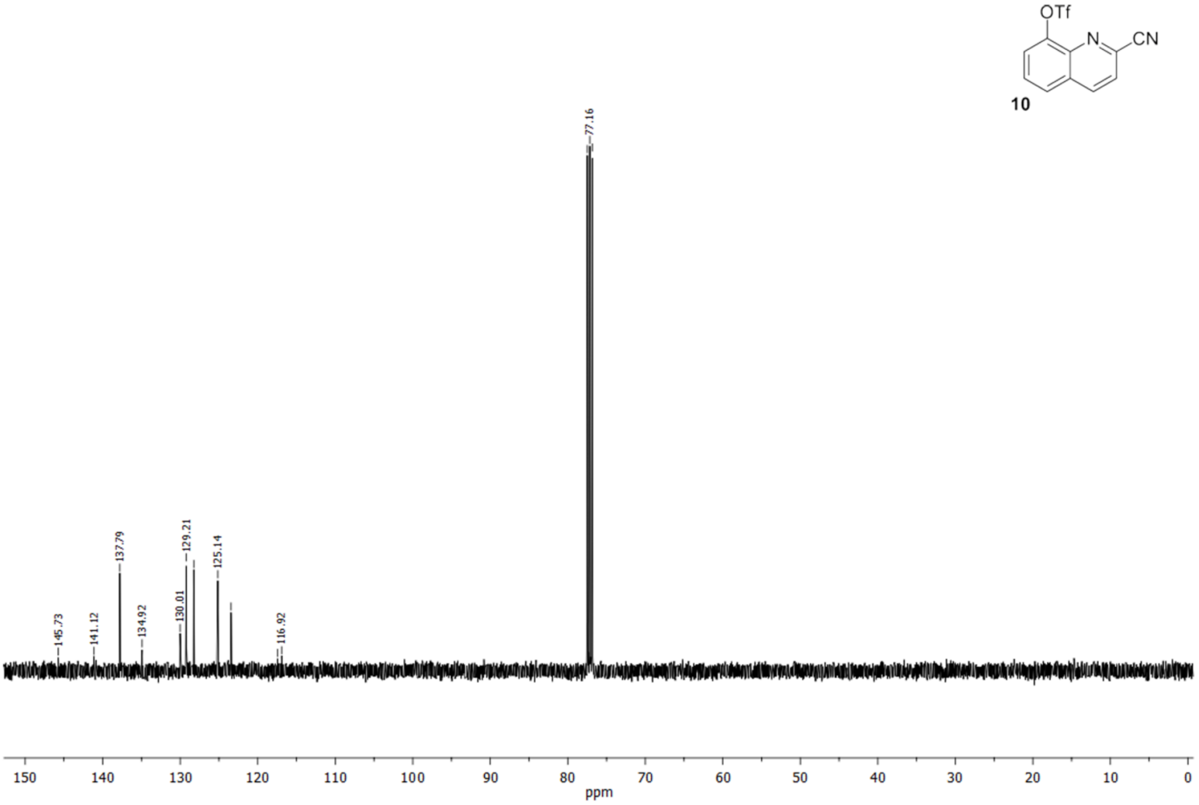

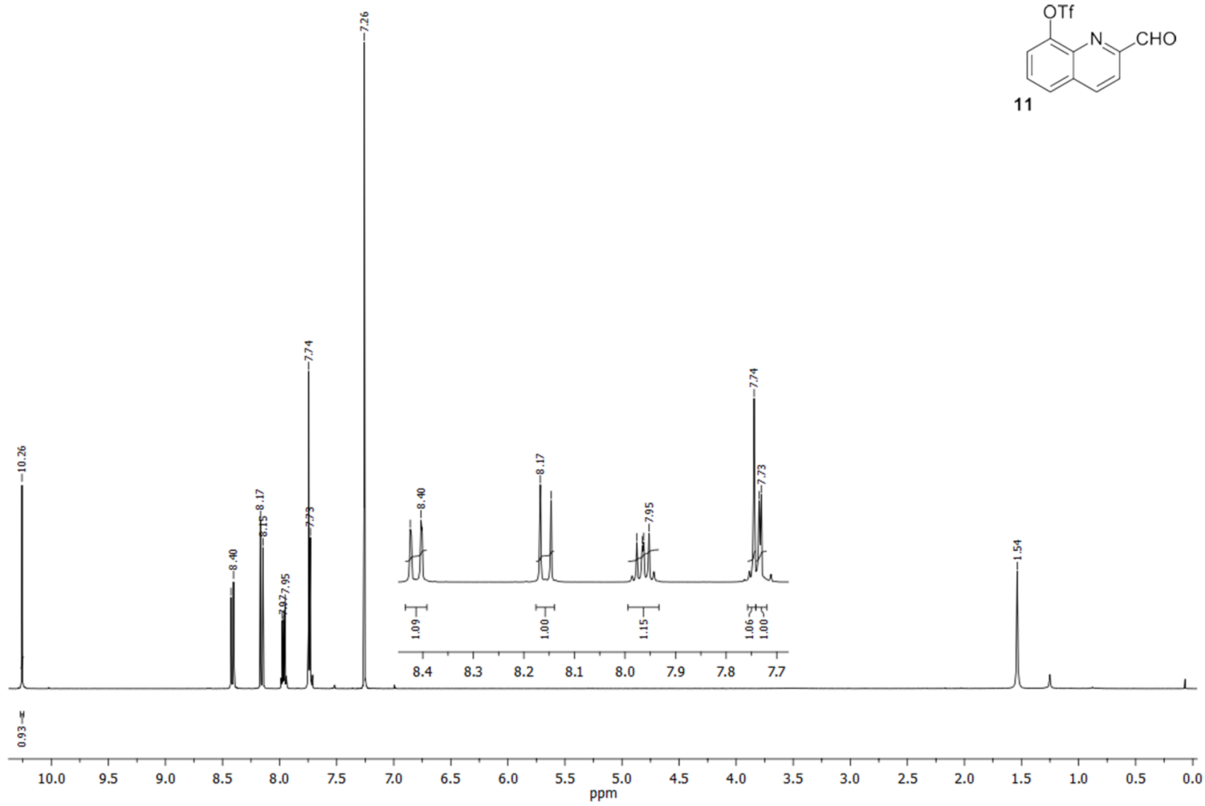
OTf
N
CHO
11
10.26
8.40
8.17
8.15
7.97
7.95
7.74
7.73
7.26
1.54
0.93
1.09
1.00
1.15
1.06
1.00
ppm

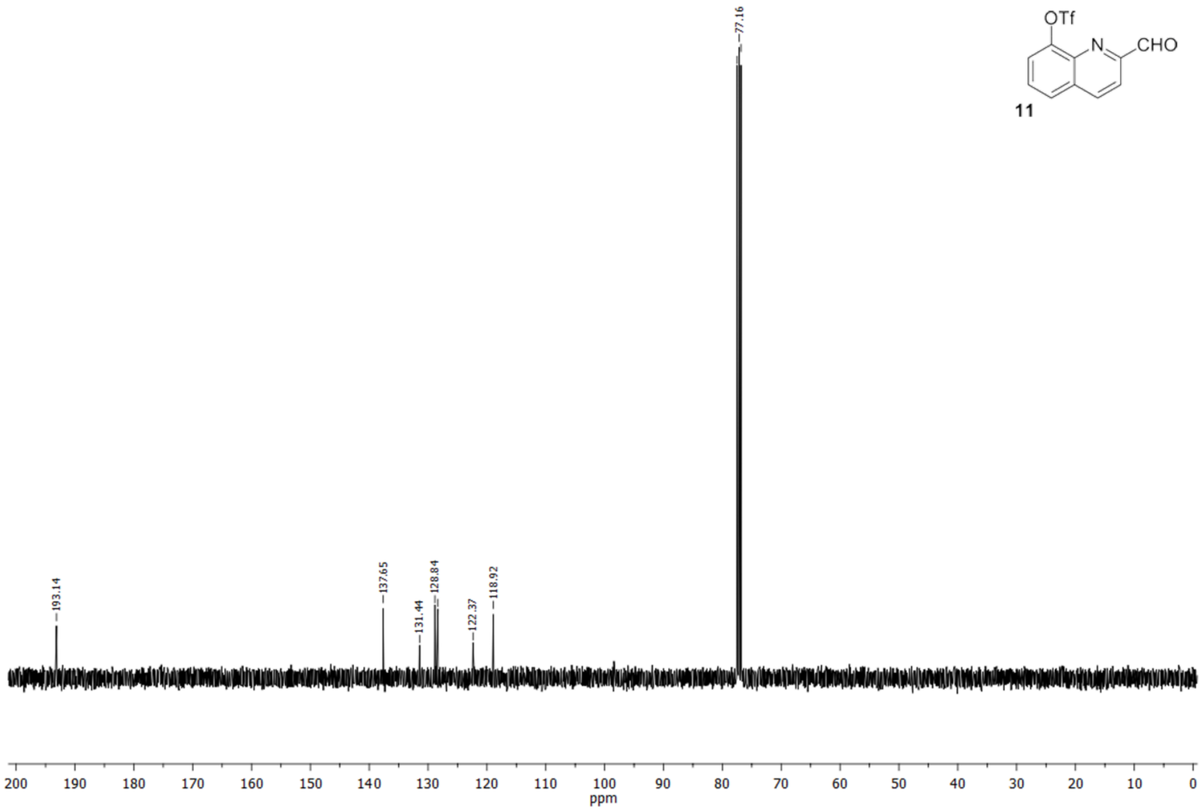
OTf
N
CHO
11
193.14
137.65
131.44
128.84
122.37
118.92
77.16
ppm

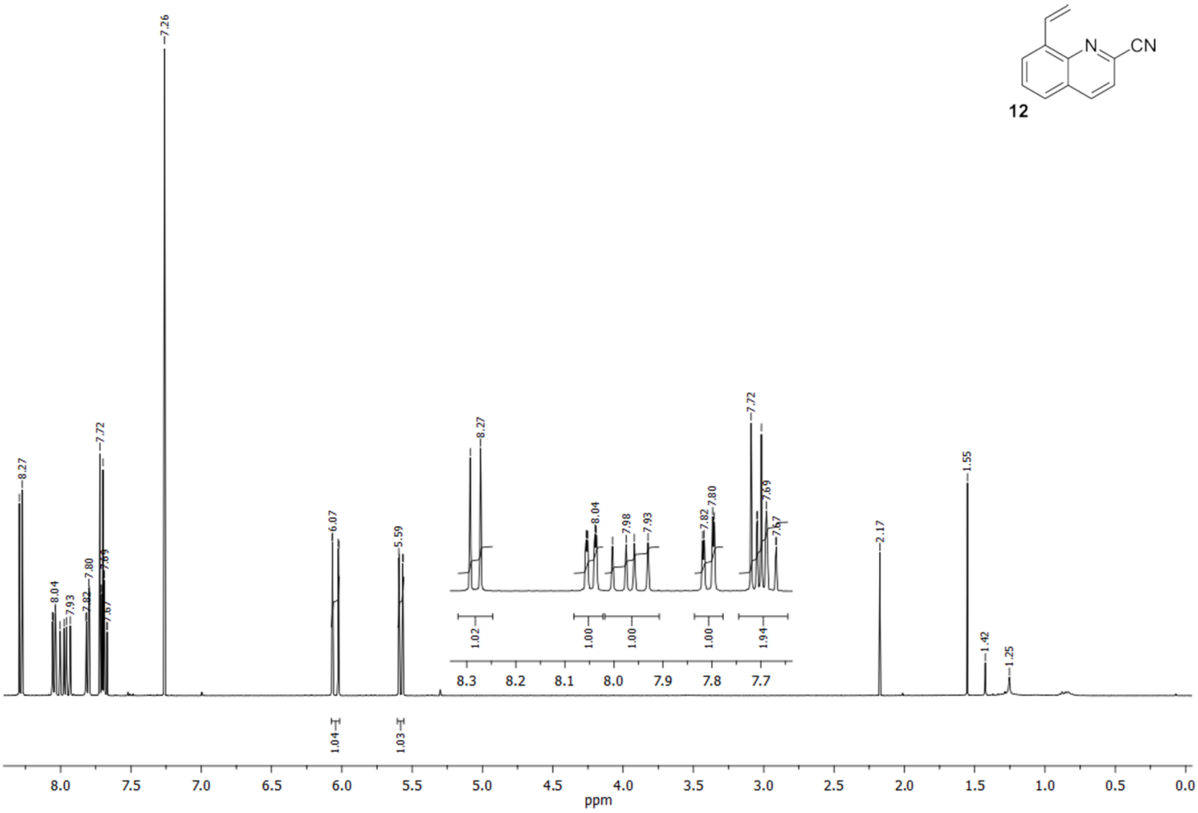
N
CN
12
7.26
8.27
8.04
7.93
7.82
7.80
7.72
7.69
7.67
6.07
5.59
2.17
1.55
1.42
1.25
1.02
1.00
1.00
1.00
1.94
1.04
1.03
ppm

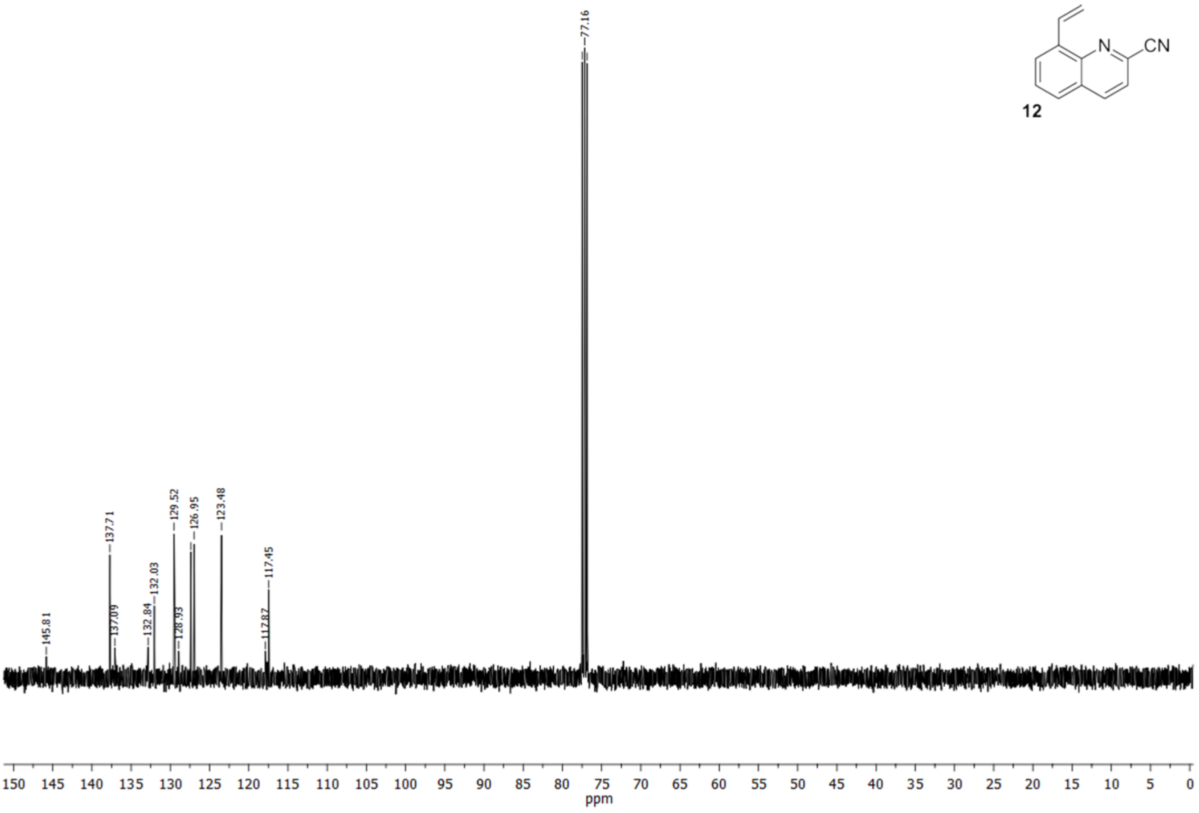
N
CN
12
77.16
145.81
137.71
137.09
132.84
132.03
129.52
128.93
126.95
123.48
117.87
117.45
ppm

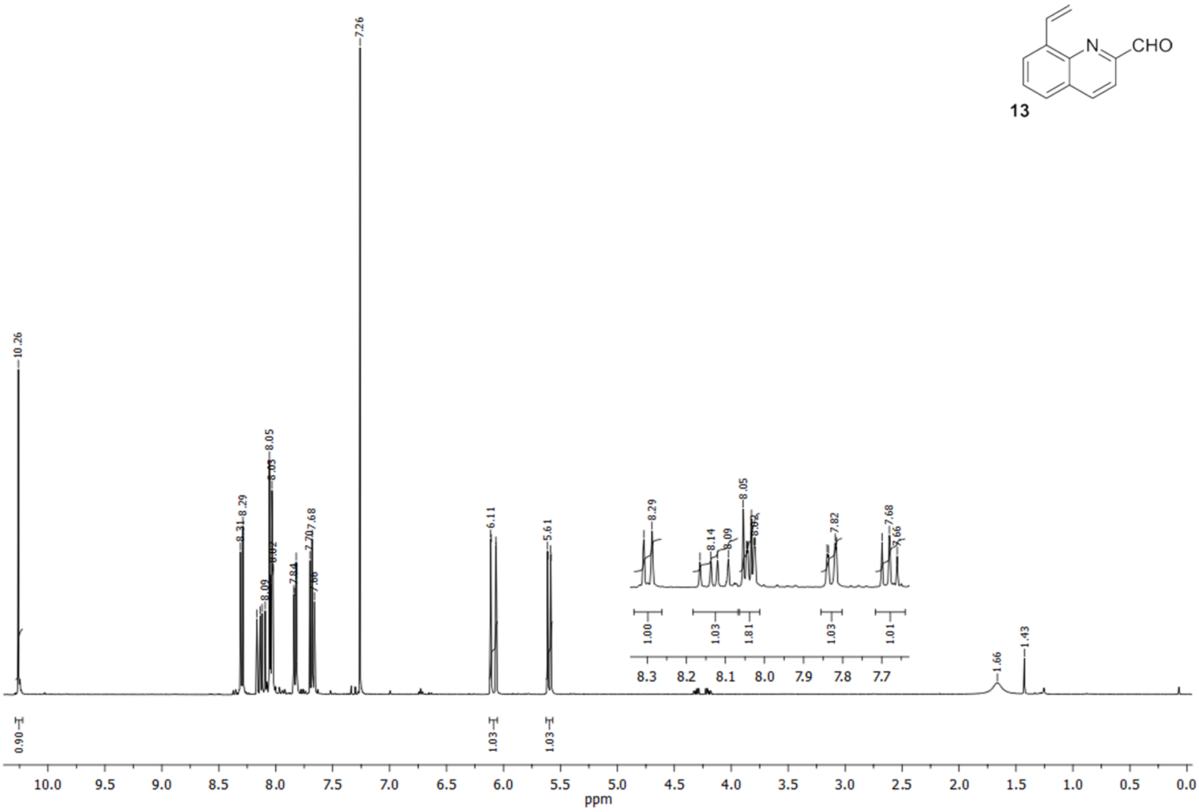
N
CHO
13
10.26
8.31
8.29
8.09
8.05
8.03
8.02
7.84
7.70
7.68
7.66
7.26
6.11
5.61
1.66
1.43
0.90
1.03
1.03
1.00
1.03
1.81
1.01
ppm

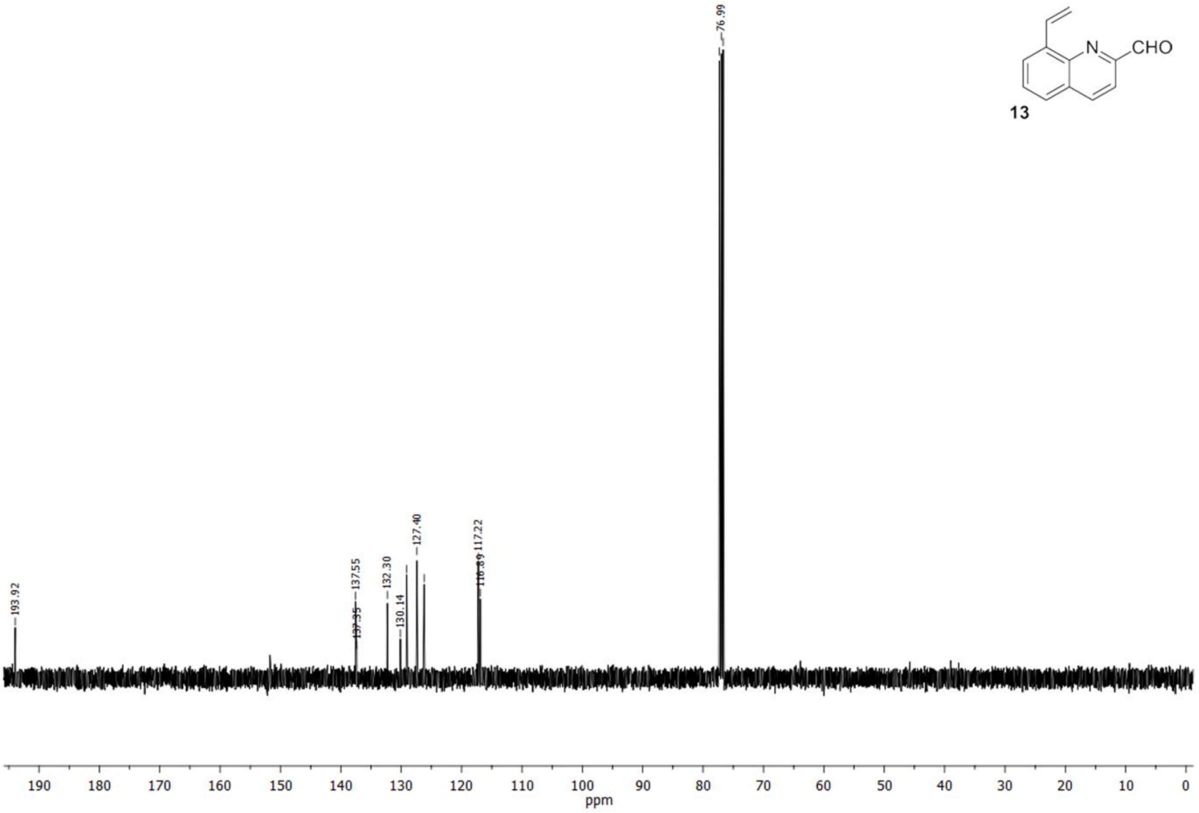
N
CHO
13
193.92
137.55
137.35
132.30
130.14
127.40
117.22
116.89
76.99
ppm

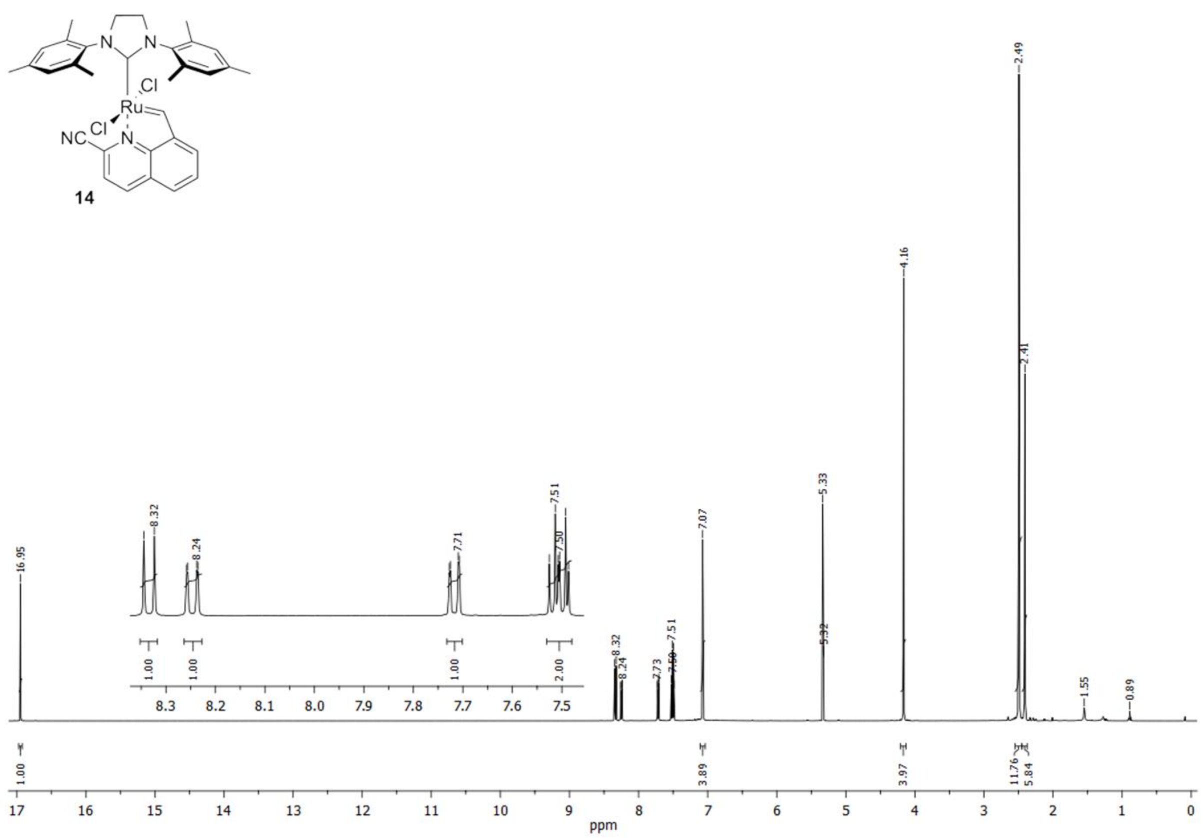
14
NC
Cl
Ru
N
16.95
8.32
8.24
7.73
7.51
7.50
7.71
7.07
5.33
5.32
4.16
2.49
2.41
1.55
0.89
1.00
3.89
3.97
11.76
5.84
2.00
ppm

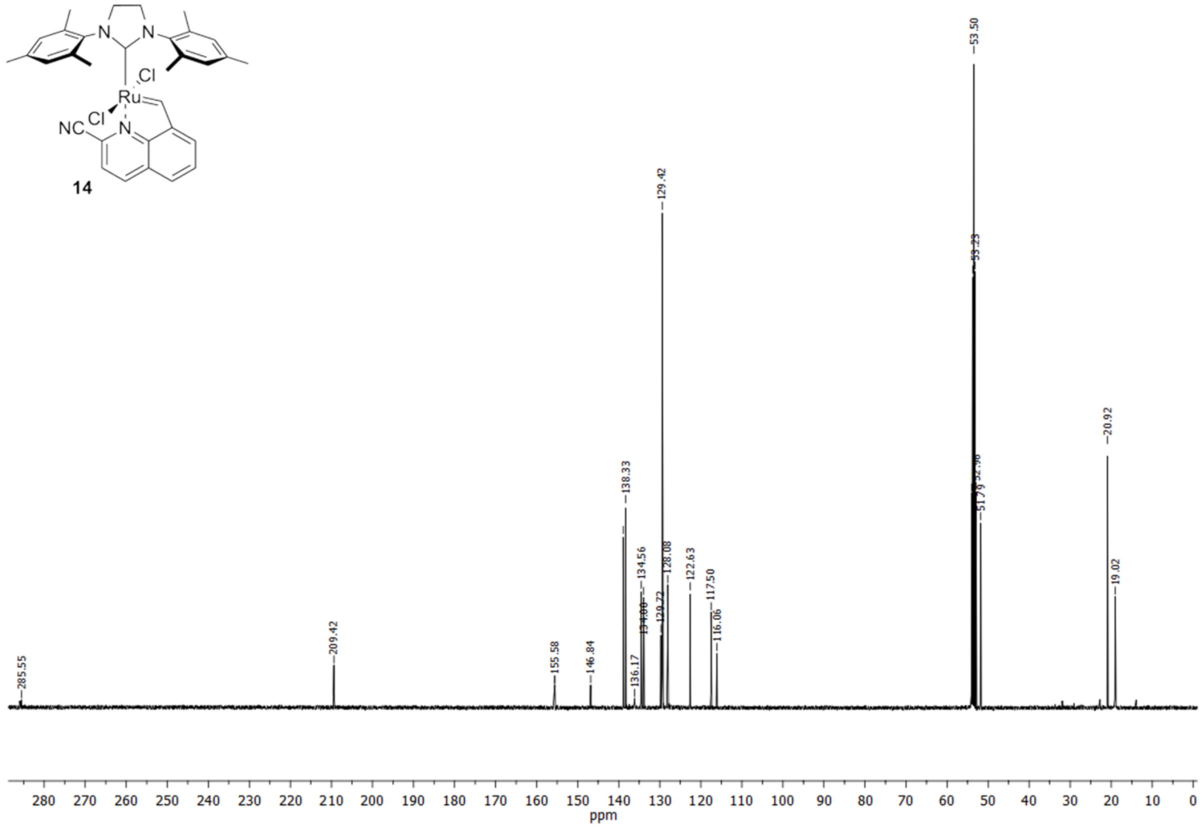
14
NC
Cl
Ru
N
285.55
209.42
155.58
146.84
138.33
136.17
134.56
134.00
129.42
129.22
128.08
122.63
117.50
116.06
53.50
53.23
20.92
19.02
ppm

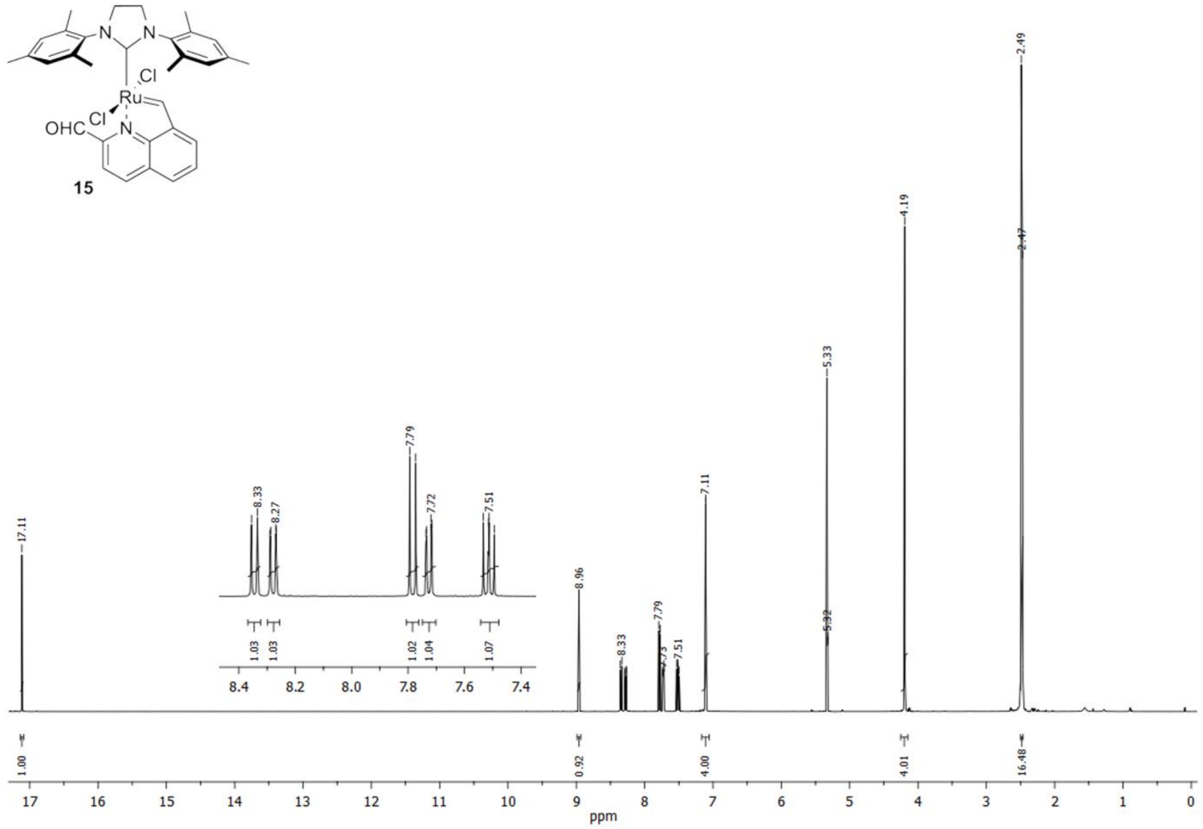
OHC
N
Ru
Cl
Cl
17.11
8.96
8.33
8.27
7.79
7.72
7.73
7.51
7.11
5.33
5.32
4.19
2.49
2.47
1.00
0.92
4.00
4.01
16.48
1.03
1.03
1.02
1.04
1.07
8.4 8.2 8.0 7.8 7.6 7.4
17 16 15 14 13 12 11 10 9 8 7 6 5 4 3 2 1 0
ppm

15

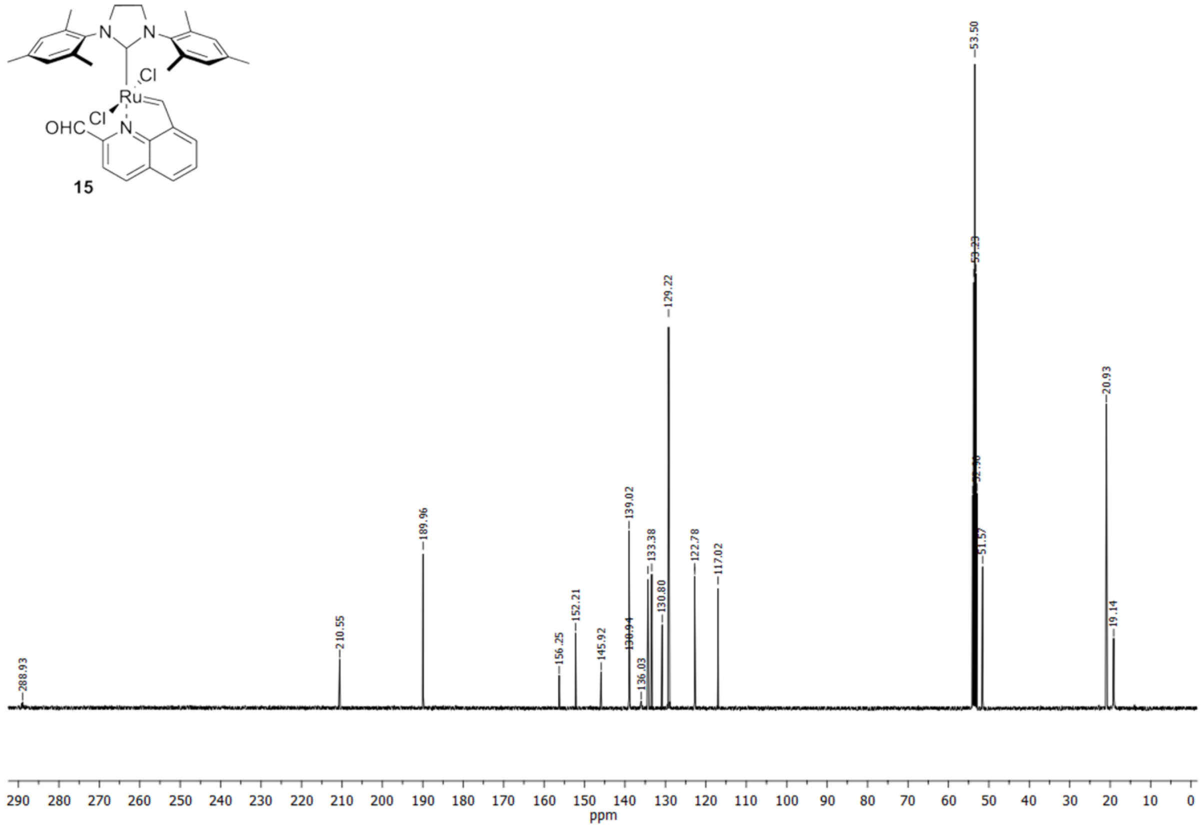
OHC
N
Ru
Cl
Cl
288.93
210.55
189.96
156.25
152.21
145.92
139.02
138.94
136.03
133.38
130.80
129.22
122.78
117.02
53.50
53.23
52.96
51.57
20.93
19.14
290 280 270 260 250 240 230 220 210 200 190 180 170 160 150 140 130 120 110 100 90 80 70 60 50 40 30 20 10 0
ppm

15

2.6. General procedure of ring-closing metathesis reactions

Diethyl diallyl malonate (0.5 mmol, 120 mg) and an internal standard – *n*-dodecane (0.15 mmol, 26 mg) were put in a Schlenk tube under Ar and dissolved in 5 mL of anhydrous toluene or xylene. The mixture was heated to the desired temperature and the examined ruthenium complex was added marking this time as a reaction start. Samples (0.2 mL) were taken and immediately quenched with 2 M ethyl-vinyl ether solution in DCM (0.2 mL). GC analysis was performed shortly after sample collection.

2.7. GC MS analyses

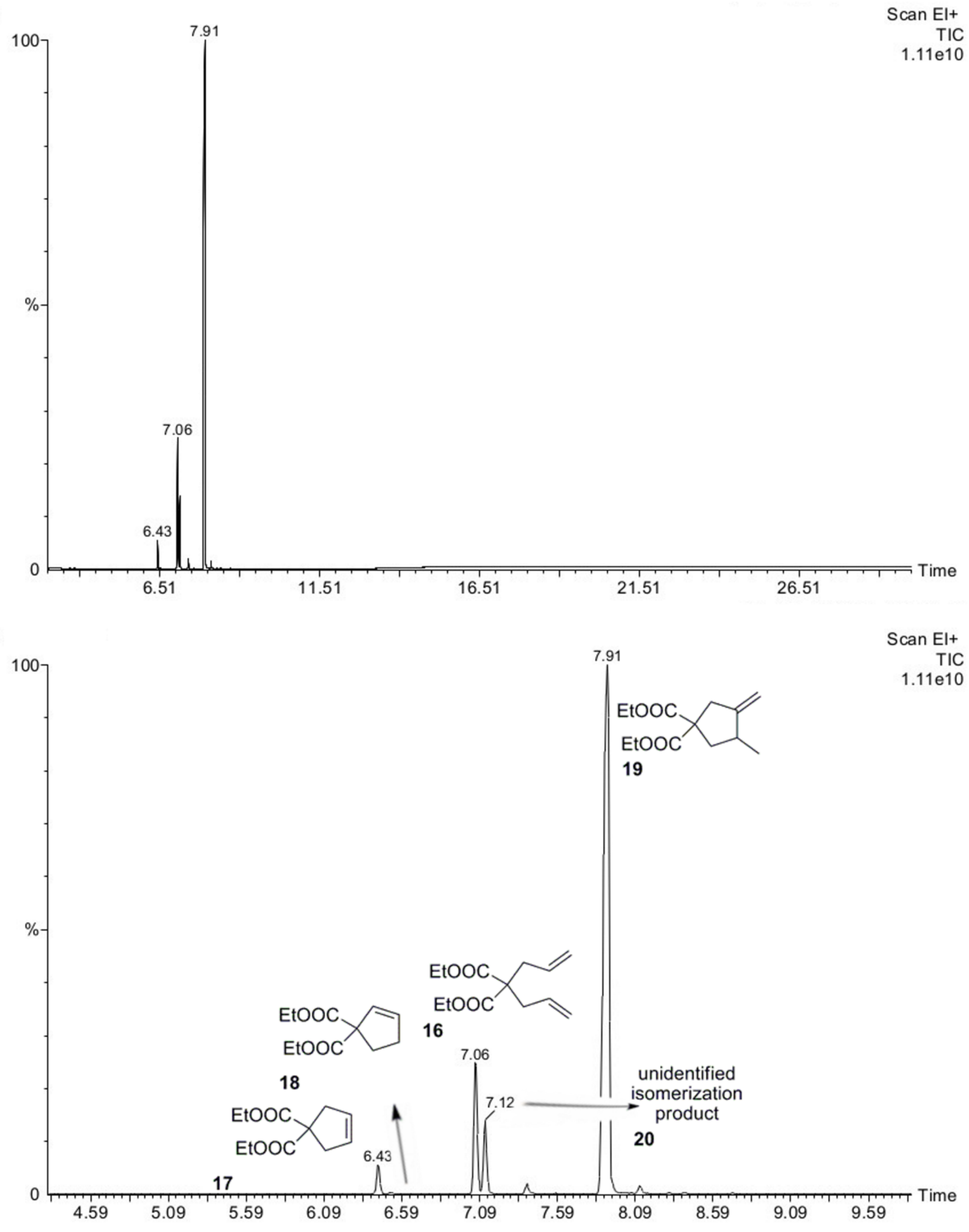

Figure 1. A selected GC of the reaction mixture after subjecting compound **16** to metathesis conditions.

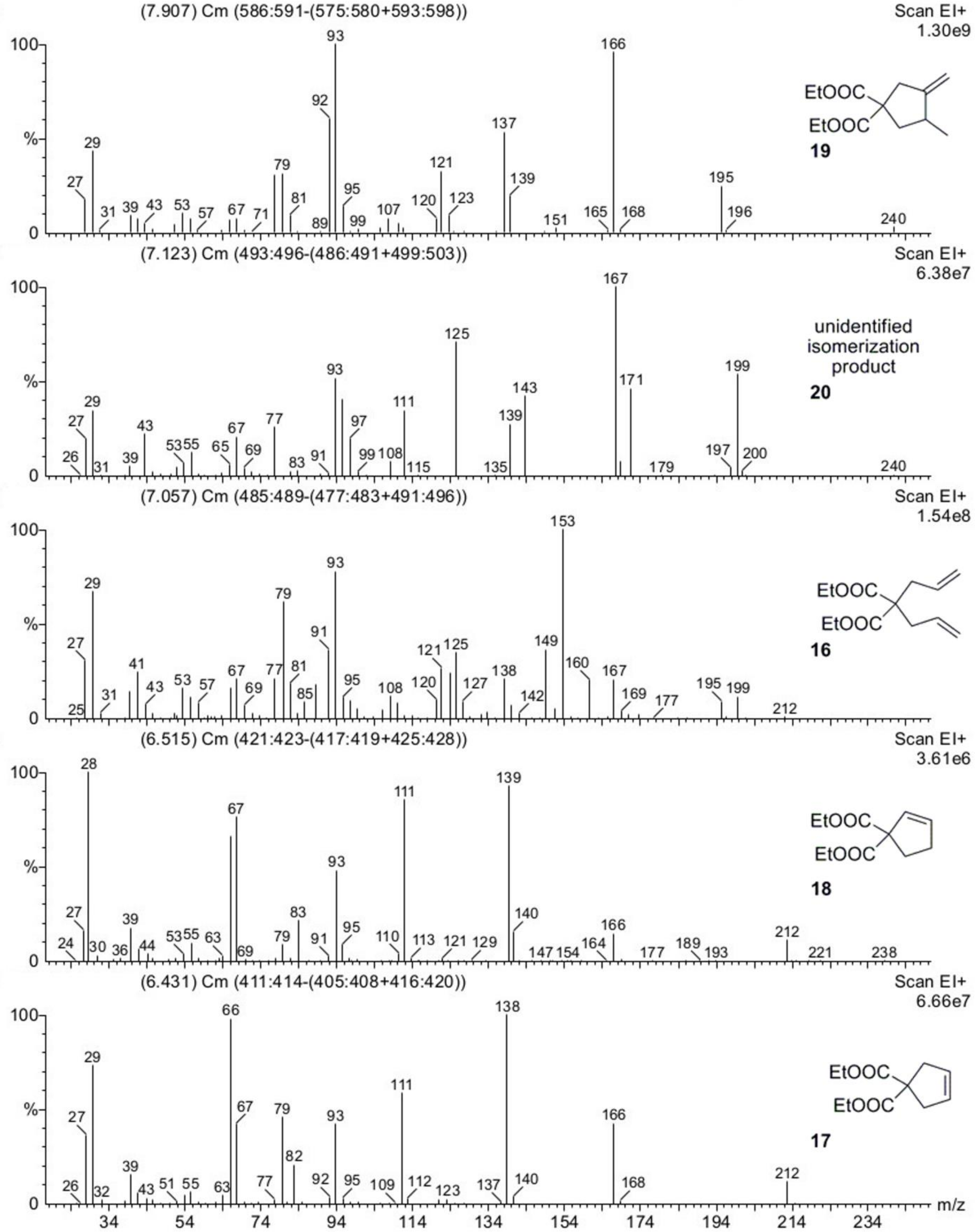

Figure 2. Mass spectra of the observed compounds.

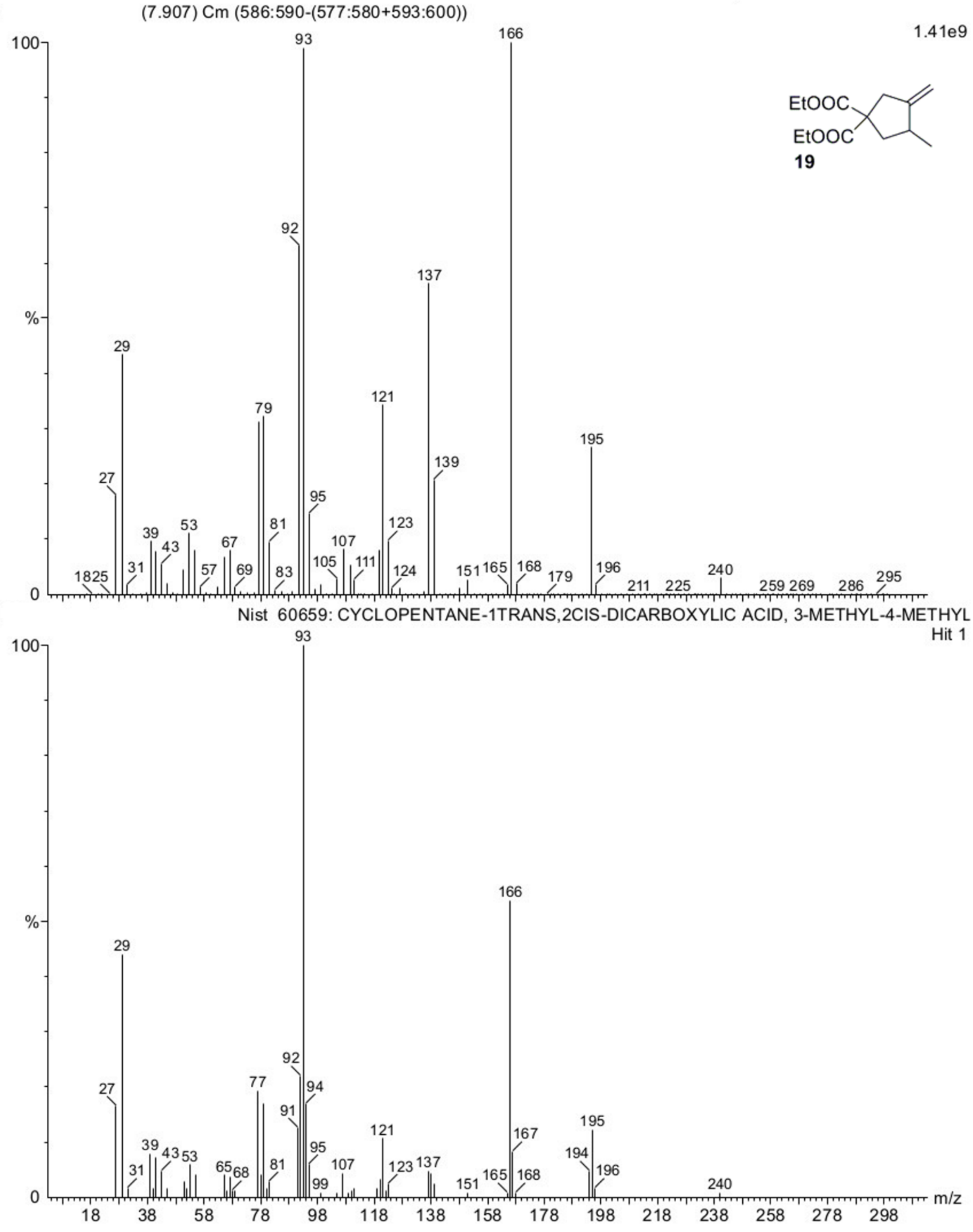

Figure 3. MS spectra comparison of the obtained compound **19** and a record from NIST database.

2.8. Simultaneous Thermal Analysis (STA)

2.8.1. Sample preparation for STA measurements with DCPD (23)

DCPD (**23**), which is solid at room temperature, was molten in a 35–40 °C water bath before use. Initiator **5a** (4.92 mg), **14** (4.88 mg) and **15** (4.88 mg) were dissolved in 600 µL dichloromethane ($c_{[Ru]}$=12.6·10^{-3} M). 60 µL of this stock solution (0.76·10^{-3} mmol) were transferred into a 2 mL glass vial and 1.0 mL of liquid *DCPD* (7.4 mmol) was added with a syringe, so that the batch was well mixed. The total content of solvent in the mixture is 7.4 w.-% (d_{DCM} = 1.33 g·mL^{-1}, d_{DCPD} = 0.98 g·mL^{-1}). The vial was immediately put into liquid nitrogen to shock-freeze the mixture. For transportation the vial was stored in a Styropfoam container. About 8–12 mg of the mixture was transferred into a cooled DSC pan, which was then subjected to the STA run. The analysis was run with a constant He gas stream of 50 mL·min^{-1} at a heat rate of 3 K·min^{-1}, starting at 20 °C.

2.8.2. Sample preparation for STA measurements with diethylester-norbornene 22

A stock solution of **5a** (4.92 mg), **14** (4.88 mg) and **15** (4.88 mg) was prepared ($c_{[Ru]}$ = 13.9·10^{-3} M). 60 µL of this stock solution (0.83·10^{-3} mmol) were transferred into a 2 mL glass vial and 100 mL of liquid **22** (d_{mon2} = 1.0 g·mL^{-1}, 0.42 mmol) were added and well mixed. The solvent was removed by a N_2-stream and then immediately shock frozen by liquid nitrogen to avoid any premature activation. For transportation the vial was stored in a Styropfoam container. About 8–12 mg of the mixture was transferred into a cooled DSC pan, which was then subjected to the STA run. The analysis was run with a constant He gas stream of 50 mL·min^{-1} at a heat rate of 3 K·min^{-1}, starting at 20 °C.

2.8.3. Polymerization of dimethylester-norbornene 21 in solution

In a Schlenk flask, **5a***,* **14** and **15** (1.27 µmol, 1.0 equiv) were dissolved in toluene (3.8 mL, c_{mon}=0.1 M). Subsequently, the mixture was heated to 80 °C then **21** (100.0 mg, 0.38 mmol, 300 eqiuv) was added, dissolved in 1 mL toluene. The reaction was followed by TLC using *c*-hexane:ethyl acetate, 3:1 (v:v) and $KMnO_4$ solution for staining. Furthermore, the progress of the reaction was monitored by sampling 200 µL of the reaction mixture and recording ^{1}H NMR spectra of the quenched sample once to twice a day. After no further conversion was observed due to very high viscosity, an excess of ethyl vinylether was added (200 µL) and stirred for 15 more minutes. Subsequently, the polymer was precipitated in cold, stirred methanol. The white solid was sampled and dried in vacuum. Characteristic polymer properties were examined by gel permeation chromatography (GPC) in THF against polystyrene standard.

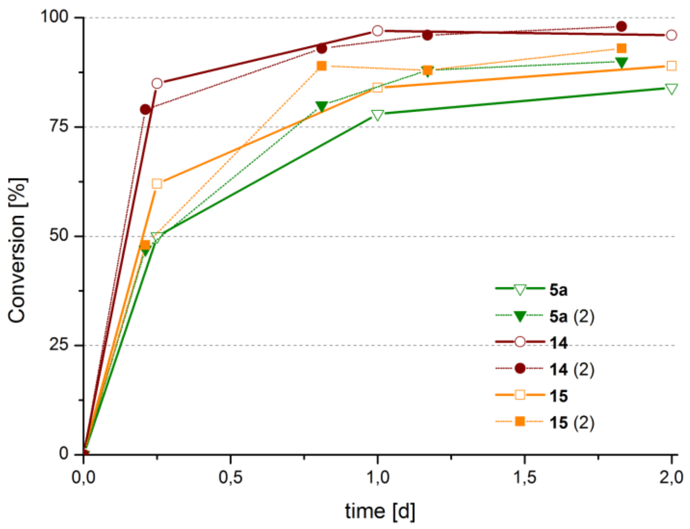

Figure 4. Time/conversion plot for the polymerization of **21** with initiators **5a**, **14** and **15**. The progress of the polymerization was determined via ^{1}H NMR.

2.9. *Trans–cis* Isomerization

2.9.1. NMR studies

In a 0.5–2 mL tube, **5a**, **14** and **15** (45.4 µmol, 1.0 equiv) were dissolved in degassed $CDCl_3$ (0.7 mL). Subsequently, the mixture was heated to 140 °C in microwave. The isomerization was monitored by ^{1}H NMR spectroscopy ($CDCl_3$, 25 °C, 300 MHz) within the carbene region.

Table 1. Experimentally observed *cis*-content, determined in $CDCl_3$ after treating catalysts in a microwave at 140 °C for 1 h. Recorded spectra on Figure 5.

catalyst	[*cis*]/([*cis*]+[*trans*]) (%)
**5a**	100
**14**	0
**15**	8

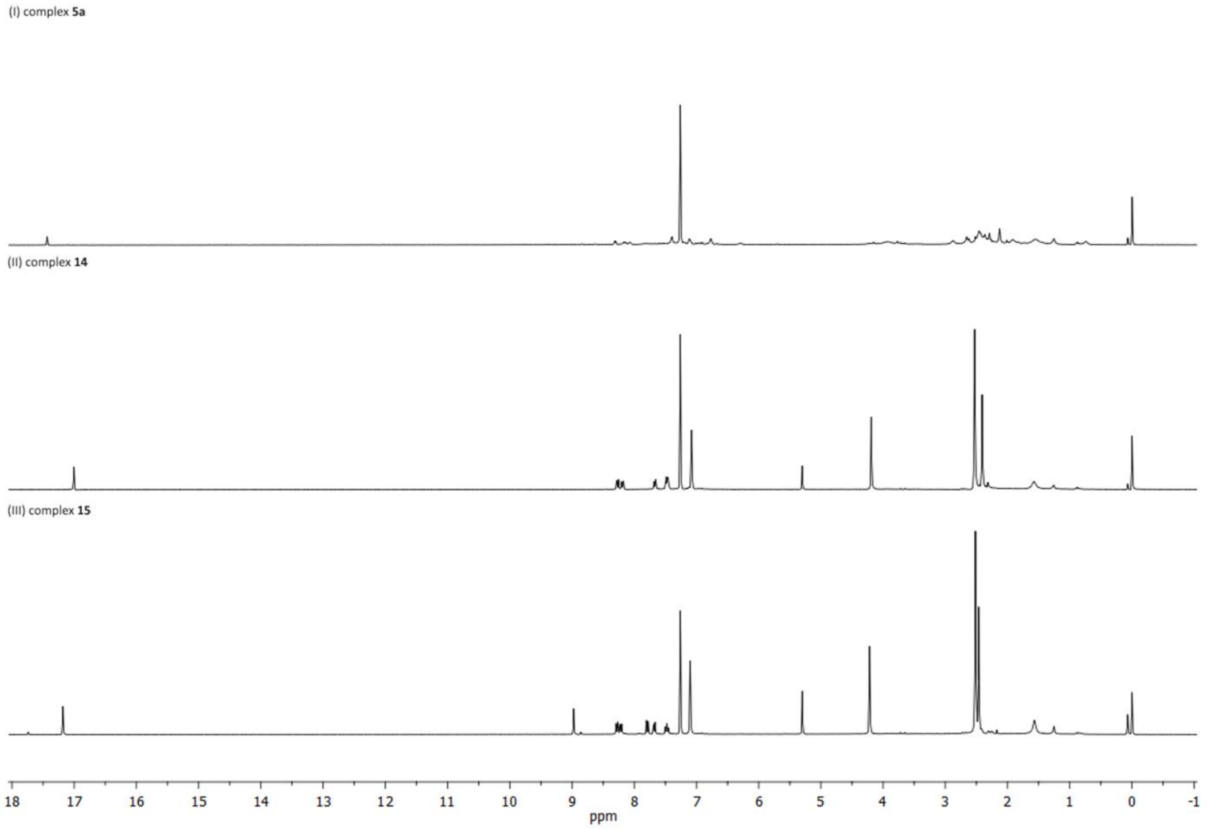

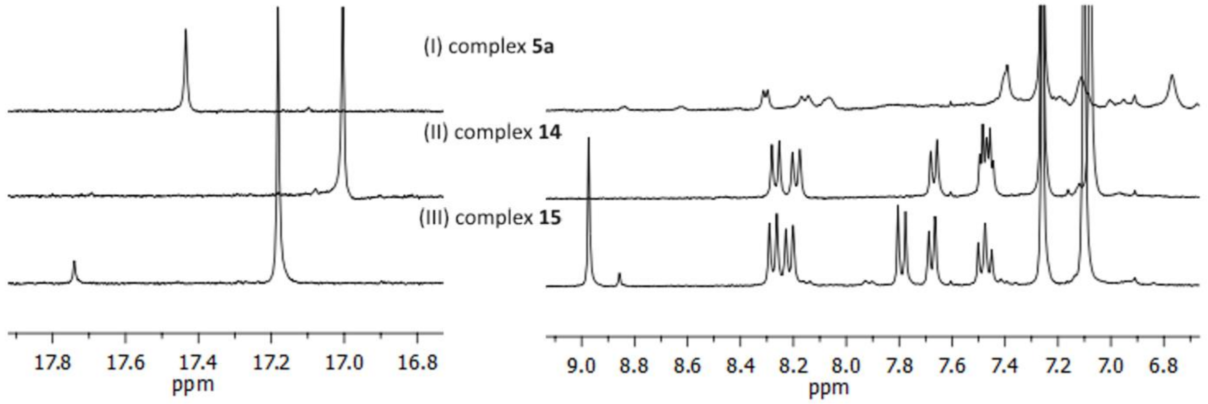

Figure 5. Studied complexes after 1 hour of microwave heating in 140 °C (top) along with magnifications of the crucial regions of spectra (bottom).

2.9.2. Preparation of *cis*-5a

Isomerization was obtained by exposing 50 mg of *trans*-**5a** complex, dissolved in 3 mL $CDCl_3$ for 30 min in a microwave at 140 °C. The raw product was identified by ^{1}H NMR spectroscopy (300 MHz) to be the desired *cis*-isomer. Traces of decomposition material and *trans*-compound were removed by precipitation with *n*-pentane and column chromatography on silica gel with CH_2Cl_2:MeOH 20:1 (v:v).

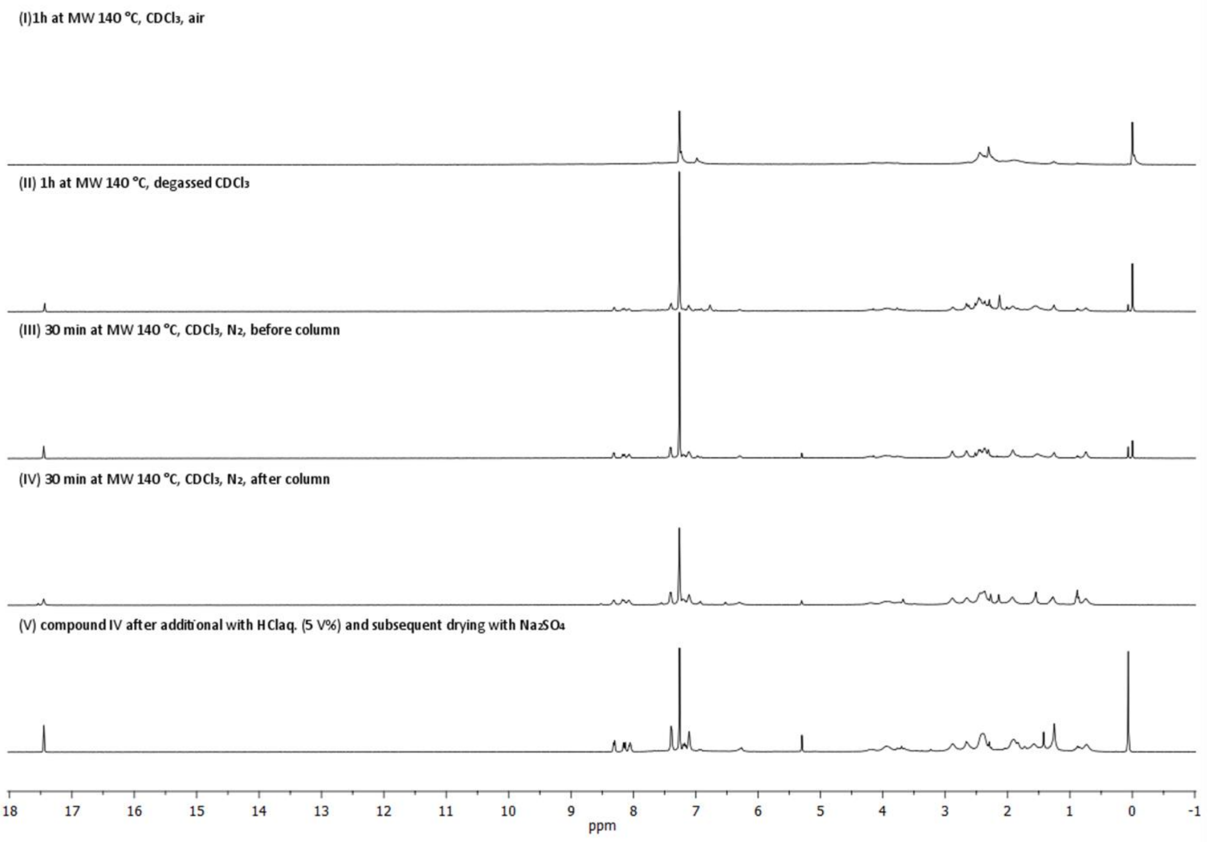

Figure 6. Preparation of ***cis*-5a**.

2.9.3. Preparation of *cis*-15

500 mg of **M31** [1,3-Bis(2,4,6-trimethylphenyl)-2-imidazolidinylidene]dichloro(3-phenyl-1*H*-inden-1-ylidene)(pyridyl)ruthenium(II) (0.668 mol, 1.0 equiv) were converted with 146 mg of the respective ligand **13** (0.801 mol, 1.2 equiv) in 10 mL of degassed, dry CH_2Cl_2 under N_2 atmosphere. After complete conversion (20 h) the reaction was terminated. The brownish reaction mixture was extracted with $HCl_{aq.}$ (5 vol %) once to get rid of pyridine, produced during the synthesis. Subsequently, the solvent of the brownish reaction mixture was removed to 2–3 mL and precipitated with *n*-pentane. The brownish precipitate was filtered, washed with *n*-pentane and dried in vacuo. Yield: 403 mg of brownish solid (94%), containing 11% of *cis*-compound.

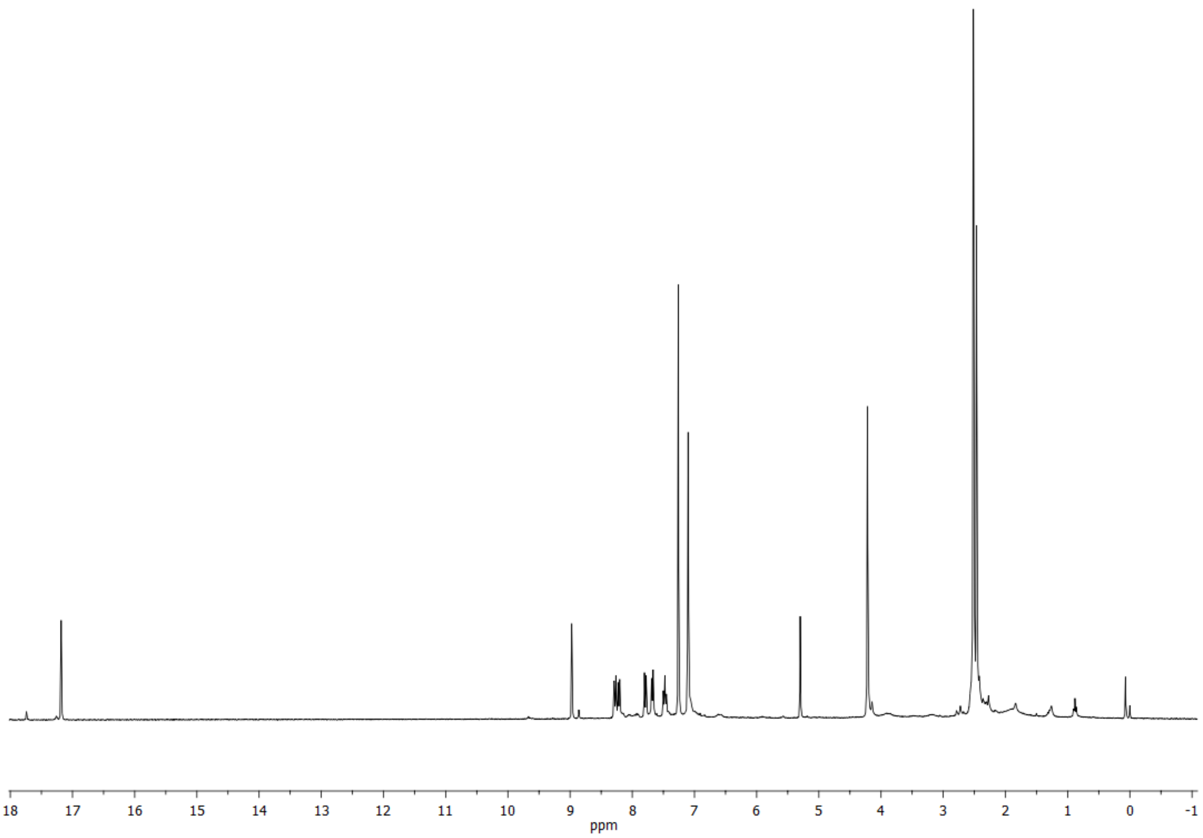

Figure 7. ^{1}H NMR (300 MHz) spectrum of *trans*- and *cis*-**15** mixture obtained by ligand exchange with **M31**.

3. X-ray crystallographic details

Diffraction data for both compounds were collected at 90(2) K by the ω-scan technique using **15**: BRUKER KAPPA APEXII ULTRA controlled by APEXII[3] software, equipped with MoKα rotating anode X-ray source (λ = 0.71073 Å, 50.0 kV, 22.0 mA) or **14**: Kuma KM4CCD κ-axis diffractometer or with graphite-monochromated Mo Kα radiation (λ = 0.71073 A, 50.0 kV, 40.0 mA). Both experiments were conducted at 100K, using the Oxford Cryostream cooling device. Crystal was mounted on a nylon loop with a droplet of Paratone-N oil and immediately cooled. Indexing, integration and scaling were performed with original software.[3] The multi-scan absorption correction was applied in the scaling procedure. Structures were solved by direct methods approach using the SHELXS-97 program and refined with the SHELXL-97 using the full-matrix least-squares procedure on F2.[4] Scattering factors incorporated in SHELXL97 were taken from Tables 4.2.6.8 and 6.1.1.4 from the International Crystallographic Tables Vol. C.[5] Details of crystal structure and data refinement are given in **Table 1**.

Non-hydrogen atoms were refined anisotropically. All hydrogen atoms were placed in the calculated positions and were treated as riding on the parent atom with U_{iso} set at 1.2 (1.5 for methyl groups)

[3] for **1**: (a) APEXII-2008v1.0 Bruker Nonius 2007 (b) SAINT V7.34A Bruker Nonius 2007 (c) SADABS-2004/1 Bruker Nonius area detector scaling and absorption correction, **2007**; for **2**: CrysAlisPro, Agilent Technologies, Version 1.171.36.20 (release 27-06-2012 CrysAlis171 .NET)
[4] G. M. Sheldrick, *Acta Crystallogr.* **1990**, *A46*, 467-473;
[5] International *Tables for Crystallography*, Ed. A., J. C. Wilson, Kluwer: Dordrecht, **1992**, Vol. C.

times U_{eq} of appropriate carrier atom. Both compounds have two molecules in the asymmetric part of the unit cell, so in the CIFiles a following numbering convention was adopted: in case of **15b** and **14b** molecules, the original numbering of **15a** and **14a** is retained with 50 added, i.e. atom N(1) in **15a** corresponds to atom N(51) in **15b**.

Crystallographic data (excluding structure factors) for the structural analysis have been deposited with the Cambridge Crystallographic Data Centre, No. CCDC- 991362 (**14**) and CCDC-991363 (**15**). Copies of this information may be obtained free of charge from: The Director, CCDC, 12 Union Road, Cambridge, CB2 1EZ, UK. Fax: +44(1223)336-033, e-mail:deposit@ccdc.cam.ac.uk, or www: www.ccdc.cam.ac.uk.

Table 2. The comparison of the geometry for investigated compounds.

	**5a-*trans***	**5a-*cis***	**15a**	**15b**	**14a**	**14b**		
Ru(1)-C(22)	1.845	1.824	1.829(5)	1.829(6)	1.842(5)	1.843(5)	Ru centre	**Bond length/ Å**
Ru(1)-C(1)	2.063	2.008	2.032(5)	2.041(5)	2.035(5)	2.048(5)		
Ru(1)-Cl(1)	2.347	2.381	2.316(1)	2.343(1)	2.320(1)	2.342(1)		
Ru(1)-Cl(2)	2.352	2.373	2.329(1)	2.345(1)	2.332(1)	2.348(1)		
Ru(1)-N(3)	2.162	2.072	2.150(4)	2.132(4)	2.151(4)	2.152(4)		
C(22)-C(23)	1.464	1.439	1.450(7)	1.440(8)	1.453(7)	1.439(7)		
N(3)-C(31)	1.327	1.330	1.334(7)	1.327(7)	1.334(7)	1.330(6)	quinoline ligand	
N(3)-C(28)	1.369	1.380	1.360(6)	1.350(7)	1.363(6)	1.366(6)		
C(31)-C(32)	-	-	1.481(7)	1.473(9)	1.437(8)	1.460(8)		
C(27)-C(28)	1.419	1.394	1.412(7)	1.416(8)	1.419(7)	1.420(7)		
C(30)-C(31)	1.406	1.398	1.415(7)	1.415(8)	1.411(7)	1.413(7)		
C(27)-C(29)	1.413	1.420	1.394(8)	1.412(9)	1.390(8)	1.421(7)		
C(30)-C(29)	1.369	1.366	1.367(7)	1.367(10)	1.371(7)	1.377(8)		
C(32)-N(4)	-	-	-	-	1.159(7)	1.132(7)		
C(32)-O(1)	-	-	1.209(7)	1.204(7)	-	-		
C(1)-N(1)-C(4)	128.6	127.3	125.7(4)	125.9(4)	125.8(4)	125.7(4)	NHC	**Plane angles/°**
C(1)-N(2)-C(13)	129.8	120.1	125.7(4)	125.4(4)	125.8(4)	126.7(4)		
C(1)-Ru(1)-C(22)	101.6	96.1	102.7(2)	102.7(2)	102.4(2)	101.3(2)		
Cl(1)- Ru(1)-Cl(2)	155.48	89.61	153.56(5)	157.53(5)	154.73(5)	158.11(5)	Ru centre	
Ru(1)-C(22)-C(23)	118.5	118.9	117.7(4)	117.8(4)	117.8(4)	118.1(4)		
Ru(1)-C(1)-N(1)-C(4)	17.7	11.2	-32.0(8)	18.1(8)	-31.9(7)	19.5(7)		**Torsion angles/°**
Ru(1)-C(1)-N(2)-C(13)	-7.2	-23.8	25.6(7)	-11.2(7)	-169.5(5)	-12.5(7)		
C(22)-Ru(1)-N(3)-C(28)	4.4	-0.6	-4.2(4)	4.1(4)	-5.1(3)	3.5(4)	Quinoline Ligand	
Ru(1)-C(22)-C(23)-C(24)	-176.5	178.4	175.7(4)	-179.2(5)	175.1(4)	-178.7(4)		
Ru(1)- N(3)-C(31)-C(32)	-	-	-4.8(7)	8.1(8)	-6.6(7)	8.3(8)		
Ru(1)- N(3)-C(31)-H(33)	2.3	2.1	-	-	-	-		

Table 3. Crystal data and refinement details

Compound	**15**	**14**
Formula	2($C_{32}H_{33}Cl_2N_3ORu$), CH_2Cl_2	2($C_{32}H_{32}Cl_2N_4Ru$), CH_2Cl_2
Formula weight	1380.09	1374.10
Crystal system	triclinic	triclinic
Space group	P-1	P-1
a(Å)	11.9466(4)	11.9668(4)
b(Å)	16.7938(6)	16.7246(5)
c(Å)	17.0469(6)	17.0884(5)
α(°)	113.163(2)	112.749(3)
b(º)	103.737(2)	103.992(3)
γ(°)	91.172(2)	91.653(3)
V($Å^3$)	3029.53(18)	3031.70(16)
Z	2	2
D_x(g cm^{-3})	1.513	1.505

F(000)	1412	1404
m(mm^{-1})	0.813	0.811
Crystal size (mm)	0.062,0.084,0.174	0.029,0.069,0.289
Q range (^{0})	1.33– 25.00	1.77– 25.00
Reflections:		
unique	10669	10685
with >2σ(I)	7309	7322
No. of parameters	207	742
R(F) [I>2σ(I)]	0.0454	0.0450
wR(F^{2}) [I>2σ(I)]	0.0982	0.0926
R(F) [all data]	0.0894	0.0877
wR(F^{2}) [all data]	0.1282	0.1219
Goodness of fit	1.038	1.056
max/min Dr (e Å^{-3})	0.812/ -0.650	0.654/ -0.642

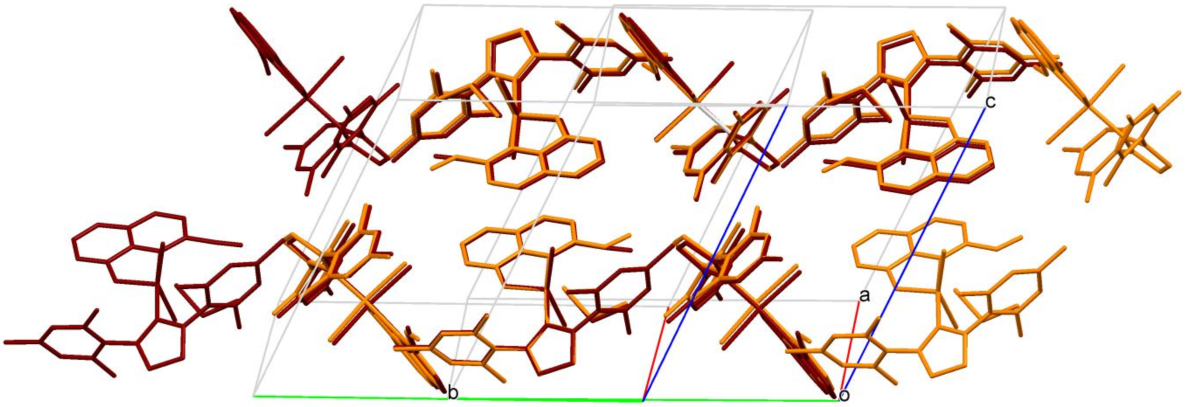

Figure 8. The superposition of units' cells for investigated structures of **14** and **15**: an example of isostructural packing.

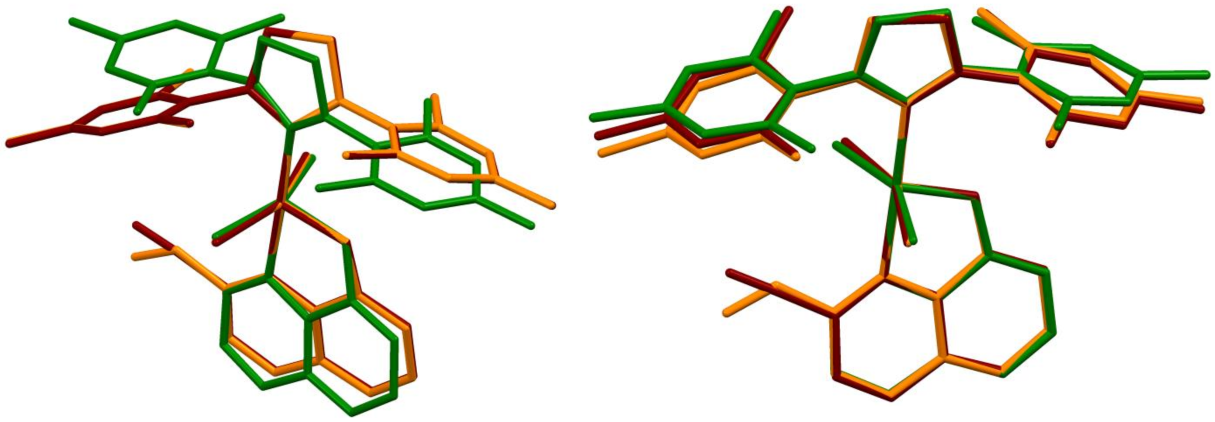

Figure 9. Superposition of *trans*-**5a**, **14** and **15**. Left: molecules of **14a** and **15a** have been used; right: molecules of **14b** and **15b** have been used. Figure 9b is present in the manuscript.

4. Details of computational approach

DFT geometry optimizations were performed at the GGA level with the Gaussian09 package[6] using the BP86 functional of Becke and Perdew.[7] The electronic configuration of the molecular systems was described with the standard splitvalence basis set with a polarization function of Ahlrichs and co-workers for H, C, N, O, and Cl (SVP keyword in Gaussian09),[8] for Ru we used the small-core, quasirelativistic Stuttgart/Dresden effective core potential, with the associated valence basis set (standard SDD keywords in Gaussian09).[9] No symmetry constraint was used and the final geometries were confirmed to be minimum or maximum potential energy structures through frequency calculations. The reported energies have been obtained through single point calculations with the M06 functional of Truhlar. In this case, the electronic configuration of the molecular systems was described by a triple-ζ basis set for main group atoms (TZVP keyword in Gaussian09).[10] Furthermore, diffuse basis sets have been incorporated for Cl and O.[11] Solvent effects including contributions of non-electrostatic terms have been estimated in single point calculations on the gas phase optimized structures, based on the polarizable continuum solvation model PCM using CH_2Cl_2 and toluene as the solvent.[12]

Table 4. Energy values (ΔE in kcal/mol), calculated in gas phase with BP86/SVP.

	trans	TS_{trans}	$14e^{-}$	TS_{cis}	TS_{conc}	cis
**14**	−7.8	21.6	11.6	29.6	34.1	0.0
**15**	−5.4	24.7	16.9	35.0	30.8	0.0

[6] Gaussian 09, Revision D.01, Frisch, M. J.; Trucks, G. W.; Schlegel, H. B.; Scuseria, G. E.; Robb, M. A.; Cheeseman, J. R.; Scalmani, G.; Barone, V.; Mennucci, B.; Petersson, G. A.; Nakatsuji, H.; Caricato, M.; Li, X.; Hratchian, H. P.; Izmaylov, A. F.; Bloino, J.; Zheng, G.; Sonnenberg, J. L.; Hada, M.; Ehara, M.; Toyota, K.; Fukuda, R.; Hasegawa, J.; Ishida, M.; Nakajima, T.; Honda, Y.; Kitao, O.; Nakai, H.; Vreven, T.; Montgomery, J. A., Jr.; Peralta, J. E.; Ogliaro, F.; Bearpark, M.; Heyd, J. J.; Brothers, E.; Kudin, K. N.; Staroverov, V. N.; Kobayashi, R.; Normand, J.; Raghavachari, K.; Rendell, A.; Burant, J. C.; Iyengar, S. S.; Tomasi, J.; Cossi, M.; Rega, N.; Millam, N. J.; Klene, M.; Knox, J. E.; Cross, J. B.; Bakken, V.; Adamo, C.; Jaramillo, J.; Gomperts, R.; Stratmann, R. E.; Yazyev, O.; Austin, A. J.; Cammi, R.; Pomelli, C.; Ochterski, J. W.; Martin, R. L.; Morokuma, K.; Zakrzewski, V. G.; Voth, G. A.; Salvador, P.; Dannenberg, J. J.; Dapprich, S.; Daniels, A. D.; Farkas, Ö.; Foresman, J. B.; Ortiz, J. V.; Cioslowski, J.; Fox, D. J. Gaussian, Inc., Wallingford CT, **2009**.

[7] a) Becke, A. D. *Phys. Rev. A* **1988**, *38*, 3098-3100; b) Perdew, J. P. *Phys. Rev. B* **1986**, *33*, 8822–8824; c) Perdew, J. P. *Phys. Rev. B* **1986**, *34*, 7406-7406.

[8] Schaefer, A.; Horn, H.; Ahlrichs, R. *J. Chem. Phys.* **1992**, *97*, 2571-2577.

[9] a) Leininger, T.; Nicklass, A.; Stoll, H.; Dolg, M.; Schwerdtfeger, P. *J. Chem. Phys.* **1996**, *105*, 1052–1059; b) Kuechle, W.; Dolg, M.; Stoll, H.; Preuss, H. *J. Chem. Phys.* **1994**, *100*, 7535-7535; c) Haeusermann, U.; Dolg, M.; Stoll, H.; Preuss, H. *Mol. Phys.* **1993**, *78*, 1211–1224.

[10] Schaefer, A.; Huber, C.; Ahlrichs, R. *J. Chem. Phys.* **1994**, *100*, 5829-5835.

[11] Rappoport, D.; Furche, F. *J. Chem. Phys*. **2010**, *133*, 134105–134111

[12] Tomasi, J.; Persico, M. *Chem. Rev.* **1994**, *94*, 2027-2094.

Table 5. Energy values (ΔG in kcal/mol), calculated in gas phase with BP86/SVP.

	trans	TS_{trans}	$14e^{-}$	TS_{cis}	TS_{conc}	cis
**14**	−8.0	18.6	9.1	27.4	33.2	0.0
**15**	−5.9	21.8	12.5	32.1	27.6	0.0

Table 6. Energy values (ΔE in kcal/mol), calculated in solvent (pcm=CH_2Cl_2) with M06/TZVP.

	trans	TS_{trans}	$14e^{-}$	TS_{cis}	TS_{conc}	cis
**14**	−1.4	36.5	28.6	21.0	36.3	0.0
**15**	2.3	32.2	28.8	22.0	37.3	0.0

Table 7. Energy values (ΔE in kcal/mol), calculated in solvent (pcm=toluene) with M06/TZVP.

	trans	cis
**5a**	1.3	0.0
**14**	−4.2	0.0
**15**	−0.7	0.0

Table 8. XYZ coordinates with solvent energies in CH_2Cl_2.

***trans*-14**: -2472.38440510 A.U.				***cis*-14**: -2472.38209886 A.U.			
Ru	-0.100325	0.432176	-0.000421	Ru	-0.237673	0.188011	1.129714
Cl	-0.616421	0.569771	-2.298520	Cl	-1.344984	1.485985	2.749038
Cl	-0.510434	0.542705	2.321015	Cl	-0.292433	-1.674864	2.623560
N	0.894315	-2.530237	0.081414	N	0.826833	-1.965825	-0.848585
N	-1.294230	-2.329672	0.050506	N	-1.369458	-1.764977	-0.861474
C	-0.122996	-1.616551	0.026460	C	-0.218965	-1.226937	-0.360112
C	0.426578	-3.934875	0.075166	C	0.387798	-3.038467	-1.771538
H	0.898793	-4.506798	0.900923	H	0.832120	-4.010852	-1.476570
H	0.712778	-4.430381	-0.879813	H	0.730657	-2.814058	-2.807081
C	-1.093450	-3.781950	0.235863	C	-1.139539	-3.009262	-1.626870
H	-1.672830	-4.356951	-0.515788	H	-1.673514	-2.970079	-2.598806
H	-1.449479	-4.090012	1.243852	H	-1.531183	-3.881401	-1.059276
C	2.308069	-2.293426	0.027957	C	2.242401	-1.755135	-0.722946
C	2.956144	-2.223123	-1.232229	C	2.884123	-0.843230	-1.602245
C	4.353224	-2.036456	-1.248159	C	4.285731	-0.726325	-1.541320
H	4.865527	-1.969854	-2.222928	H	4.786169	-0.011864	-2.216576
C	5.108105	-1.925513	-0.062148	C	5.060961	-1.496937	-0.650750
C	4.426373	-1.994445	1.170179	C	4.392769	-2.406089	0.193098
H	4.995997	-1.895646	2.109650	H	4.979996	-3.022657	0.894331
C	3.031105	-2.181108	1.243060	C	2.992380	-2.568365	0.168883
C	2.168310	-2.287187	-2.519921	C	2.086486	-0.013279	-2.580562
H	2.838692	-2.212209	-3.398248	H	2.751552	0.610760	-3.208443
H	1.423657	-1.463588	-2.579398	H	1.375996	0.659055	-2.056374
H	1.600261	-3.237480	-2.613655	H	1.477466	-0.646076	-3.260977
C	6.611980	-1.767247	-0.111590	C	6.569151	-1.381056	-0.632814
H	7.115836	-2.758966	-0.135654	H	7.029178	-2.056049	-1.388251
H	6.998049	-1.228913	0.777665	H	6.990576	-1.662063	0.353407
H	6.937150	-1.217354	-1.018152	H	6.905387	-0.351729	-0.872692

C	2.317443	-2.208054	2.574358	C	2.319649	-3.582105	1.061335
H	1.576239	-1.382275	2.651008	H	1.466580	-3.132917	1.616596
H	3.035603	-2.111875	3.411978	H	3.037411	-4.005475	1.791019
H	1.753749	-3.154249	2.722337	H	1.913639	-4.434014	0.471433
C	-2.638738	-1.806505	0.013701	C	-2.714536	-1.253679	-0.769938
C	-3.296256	-1.696379	-1.243598	C	-3.137600	-0.323476	-1.757850
C	-4.597821	-1.161897	-1.266623	C	-4.476579	0.099411	-1.751369
H	-5.102044	-1.046202	-2.240728	H	-4.804012	0.835928	-2.503354
C	-5.269253	-0.771173	-0.091332	C	-5.400172	-0.368916	-0.795138
C	-4.631400	-1.003778	1.140555	C	-4.957590	-1.319303	0.142130
H	-5.160380	-0.758232	2.076754	H	-5.669916	-1.715494	0.884947
C	-3.331636	-1.540643	1.225288	C	-3.631136	-1.799412	0.167202
C	-2.765772	-1.899806	2.579724	C	-3.240437	-2.891023	1.132815
H	-1.661384	-1.875113	2.596410	H	-2.268590	-2.682502	1.628313
H	-3.106779	-2.916949	2.879624	H	-3.154420	-3.872149	0.613268
H	-3.120660	-1.194979	3.357118	H	-4.009757	-3.010262	1.920725
C	-2.646052	-2.169747	-2.521955	C	-2.173205	0.204745	-2.795233
H	-2.295117	-3.221210	-2.440083	H	-1.720783	-0.611020	-3.399949
H	-1.767742	-1.541074	-2.778913	H	-1.328538	0.753981	-2.327756
H	-3.362915	-2.120777	-3.365012	H	-2.683581	0.895913	-3.493803
C	-6.618907	-0.097176	-0.152254	C	-6.810256	0.170774	-0.760832
H	-7.191574	-0.396692	-1.053562	H	-7.491618	-0.489097	-0.187480
H	-6.481746	1.005648	-0.193687	H	-7.225972	0.299688	-1.781658
H	-7.233493	-0.320367	0.743706	H	-6.823438	1.171075	-0.275263
N	-0.199717	2.575185	0.008148	N	-0.017464	2.001559	0.106303
C	1.717269	0.805465	-0.040471	C	1.591974	0.352033	1.300405
C	3.437018	2.698222	-0.065978	C	3.578113	1.885421	0.783608
C	2.123475	2.199502	-0.041378	C	2.221795	1.529769	0.724402
C	1.043422	3.141925	-0.014016	C	1.302286	2.422325	0.088881
C	1.267777	4.554865	-0.011859	C	1.732849	3.674392	-0.449405
C	2.619574	5.013554	-0.037104	C	3.126113	3.984137	-0.393580
H	2.819319	6.096794	-0.035818	H	3.475820	4.940674	-0.813368
C	3.673065	4.097604	-0.063383	C	4.024154	3.103144	0.208916
H	4.710562	4.467268	-0.082918	H	5.093112	3.364316	0.256826
C	0.111023	5.391060	0.014963	C	0.728844	4.545844	-0.968737
H	0.222805	6.486828	0.018110	H	1.007679	5.518892	-1.402223
C	-1.148895	4.810859	0.036246	C	-0.598602	4.163311	-0.874394
H	-2.062783	5.421512	0.056362	H	-1.411455	4.820145	-1.215522
C	-1.274797	3.386385	0.031693	C	-0.950047	2.895456	-0.311756
H	2.520391	0.045790	-0.063632	H	2.193371	-0.331647	1.934461
C	-2.589386	2.797011	0.051627	C	-2.352511	2.608648	-0.184164
N	-3.702725	2.422802	0.068333	H	4.291547	1.211840	1.283080
H	4.283305	1.993421	-0.087281	N	-3.526508	2.550549	-0.162718
**TS1$_{trans-14}$**: -2472.38424061 A.U.				**TS1$_{cis-14}$**: -2472.32660930 A.U.			
C	0.122067	-1.615044	0.028979	C	-0.909407	-1.206488	0.432338
C	-0.428496	-3.932874	0.085016	C	-0.892166	-2.883374	2.098103
H	-0.715094	-4.431000	-0.868448	H	-0.381573	-2.871635	3.083545
H	-0.900876	-4.502104	0.912517	H	-0.969054	-3.943879	1.770718
C	1.091551	-3.780214	0.245035	C	-2.261911	-2.177579	2.121761
H	1.447550	-4.085059	1.254004	H	-3.111884	-2.883367	2.016081
H	1.670618	-4.357976	-0.504742	H	-2.424621	-1.591404	3.054794
C	-3.036334	-2.184066	1.243491	C	1.687288	-3.446914	0.084810
C	-4.432918	-2.010106	1.167316	C	3.067137	-3.720258	-0.011847
H	-5.006755	-1.923441	2.105481	H	3.394916	-4.538883	-0.674665
C	-5.111940	-1.945141	-0.066831	C	4.031279	-2.995872	0.716989
C	-4.354138	-2.055217	-1.250705	C	3.581679	-1.980765	1.585062
H	-4.866115	-2.003057	-2.226508	H	4.314714	-1.421412	2.190636
C	-2.955308	-2.229370	-1.231480	C	2.215951	-1.662329	1.718170
C	-2.309446	-2.291547	0.030054	C	1.277148	-2.383182	0.932508

C	-2.325943	-2.212120	2.576535
H	-3.045851	-2.114537	3.412490
H	-1.583701	-1.387454	2.654398
H	-1.764000	-3.159140	2.726329
C	-6.609876	-1.736133	-0.119546
H	-7.116670	-2.185454	0.758550
H	-7.052258	-2.176275	-1.036080
H	-6.865336	-0.653287	-0.122576
C	-2.165180	-2.297683	-2.517539
H	-2.833832	-2.224045	-3.397298
H	-1.597798	-3.248733	-2.608259
H	-1.419880	-1.474791	-2.577378
C	2.637880	-1.806656	0.014487
C	3.333035	-1.538127	1.224216
C	4.633261	-1.002999	1.135857
H	5.164042	-0.755431	2.070500
C	5.269464	-0.774757	-0.097711
C	4.595698	-1.168037	-1.270781
H	5.098551	-1.055809	-2.246003
C	3.293503	-1.700883	-1.244142
C	2.769053	-1.892881	2.580584
H	3.126220	-1.186405	3.355446
H	3.109328	-2.909604	2.882724
H	1.664725	-1.866752	2.599119
C	6.619987	-0.102829	-0.162447
H	7.188753	-0.401561	-1.066462
H	7.237592	-0.328807	0.730759
H	6.484646	1.000321	-0.201079
C	2.640647	-2.176752	-2.520218
H	3.357339	-2.134461	-3.363781
H	1.765084	-1.545118	-2.779301
H	2.284842	-3.226175	-2.433908
C	-1.716743	0.807820	-0.044459
H	-2.519824	0.048094	-0.070116
C	-2.122094	2.202120	-0.045562
C	-3.435137	2.702018	-0.072498
H	-4.281942	1.997962	-0.095971
C	-3.670360	4.101496	-0.069475
H	-4.707556	4.471903	-0.090819
C	-2.616306	5.016711	-0.040519
H	-2.815316	6.100079	-0.038891
C	-1.264921	4.557012	-0.012939
C	-0.107676	5.392455	0.016698
H	-0.218781	6.488288	0.020397
C	1.151821	4.811451	0.040022
H	2.066052	5.421511	0.062334
C	1.276812	3.386904	0.034748
C	-1.041498	3.143921	-0.015553
N	-0.895681	-2.527941	0.087114
N	1.292986	-2.328635	0.054811
N	0.201245	2.576425	0.008662
Cl	0.506088	0.544065	2.322004
Cl	0.621971	0.570534	-2.296916
Ru	0.100515	0.433598	-0.000178
C	2.590965	2.796667	0.056694
N	3.704021	2.421723	0.075117

14e^{-}-14: -2472.35093467 A.U.

C	-1.333549	1.058362	0.095955
C	-1.503931	3.415903	-0.077176
H	-1.292586	3.841187	-1.085313

C	0.690193	-4.268542	-0.695651
H	1.206275	-5.013743	-1.332119
H	0.057144	-3.628469	-1.348771
H	0.002534	-4.827979	-0.024348
C	5.507065	-3.291871	0.568429
H	6.045647	-3.181031	1.531962
H	5.983648	-2.589520	-0.150721
H	5.683863	-4.318737	0.189987
C	1.764303	-0.582828	2.670239
H	2.599488	-0.247278	3.315549
H	0.941742	-0.925341	3.332154
H	1.390490	0.299704	2.107338
C	-3.258211	-0.410461	0.575720
C	-4.248128	-0.890235	-0.325188
C	-5.307906	-0.024790	-0.662709
H	-6.075309	-0.382827	-1.369397
C	-5.419712	1.270796	-0.121146
C	-4.445512	1.692593	0.805961
H	-4.525811	2.695173	1.257970
C	-3.363024	0.874554	1.178144
C	-4.180380	-2.283311	-0.902531
H	-5.062669	-2.487966	-1.539994
H	-4.153581	-3.059552	-0.107621
H	-3.264790	-2.413996	-1.519899
C	-6.542350	2.194749	-0.535297
H	-6.898666	2.812647	0.314348
H	-7.406802	1.634366	-0.944603
H	-6.200237	2.898319	-1.326018
C	-2.342537	1.362583	2.178097
H	-2.664054	2.314600	2.641685
H	-1.364862	1.545667	1.685194
H	-2.168581	0.631284	2.994790
C	1.192132	-0.150448	-1.203974
H	1.558950	-1.160209	-1.503464
C	2.241894	0.874658	-1.343865
C	3.083045	0.729784	-2.461662
H	2.974196	-0.172741	-3.085067
C	4.010927	1.729916	-2.845764
H	4.617438	1.586209	-3.753655
C	4.146123	2.885889	-2.088144
H	4.855169	3.676171	-2.383334
C	3.378957	3.053355	-0.898072
C	3.517802	4.198998	-0.060178
H	4.212619	5.001994	-0.354782
C	2.791325	4.287523	1.114111
H	2.869322	5.155499	1.784992
C	1.941967	3.187999	1.454904
C	2.433139	2.033240	-0.493366
N	-0.121210	-2.100547	1.105503
N	-2.175456	-1.281843	0.955764
N	1.767305	2.111970	0.688476
Cl	-1.044692	-1.749624	-2.563464
Cl	-0.875725	2.369985	-1.009368
Ru	-0.637977	0.035408	-1.054838
C	1.229547	3.215062	2.716768
N	0.668856	3.257494	3.747606

TS2-14: -2472.35093467 A.U.

C	-0.554563	-1.337713	0.107200
C	-0.288802	-3.624029	0.757464
H	0.172885	-3.989028	1.699635

H	-1.305146	4.209995	0.670859
C	-2.916394	2.826105	0.029529
H	-3.392893	3.052538	1.010506
H	-3.599779	3.177192	-0.770579
C	1.341626	2.726574	1.553764
C	2.716392	3.028932	1.617991
H	3.168393	3.216928	2.606702
C	3.524459	3.097911	0.464960
C	2.925585	2.838970	-0.784436
H	3.546466	2.860222	-1.694856
C	1.556671	2.528436	-0.904269
C	0.775133	2.473221	0.278915
C	0.503364	2.658375	2.808648
H	1.116201	2.873848	3.705738
H	0.041265	1.655952	2.933228
H	-0.329882	3.394219	2.789121
C	4.989629	3.460992	0.561322
H	5.444389	3.079914	1.498579
H	5.125805	4.565319	0.560426
H	5.565672	3.059451	-0.296184
C	0.954624	2.208421	-2.253822
H	1.720919	2.261959	-3.050742
H	0.137442	2.910647	-2.526576
H	0.516903	1.187271	-2.267185
C	-3.756182	0.476339	-0.355707
C	-4.594330	0.043947	0.706131
C	-5.666594	-0.818091	0.394751
H	-6.310349	-1.173059	1.217175
C	-5.947664	-1.222294	-0.923661
C	-5.139439	-0.711830	-1.959737
H	-5.364363	-0.984267	-3.004622
C	-4.050573	0.141625	-1.706459
C	-4.410626	0.533724	2.123516
H	-4.941658	-0.124060	2.839493
H	-4.834859	1.555566	2.246310
H	-3.344186	0.565508	2.421670
C	-7.076406	-2.183010	-1.222651
H	-7.568051	-1.949748	-2.189380
H	-7.849328	-2.169310	-0.428025
H	-6.697491	-3.226370	-1.295615
C	-3.228736	0.690615	-2.848315
H	-3.726725	0.500691	-3.819258
H	-2.230021	0.206382	-2.876392
H	-3.064659	1.784696	-2.754406
C	1.063634	-0.655694	0.292152
H	1.592449	0.220336	-0.125490
C	1.908317	-1.805349	0.605051
C	1.496168	-2.850119	1.455420
H	0.514904	-2.772879	1.951755
C	2.321948	-3.968259	1.725255
H	1.952921	-4.756779	2.399771
C	3.587060	-4.075500	1.156164
H	4.228181	-4.947715	1.362485
C	4.071190	-3.045683	0.298700
C	5.359602	-3.090920	-0.311035
H	6.016423	-3.955345	-0.121397
C	5.775222	-2.054537	-1.130725
H	6.762666	-2.055462	-1.615199
C	4.875302	-0.960841	-1.332637
C	3.238156	-1.894645	0.016409

H	-0.140737	-4.412461	-0.012753
C	-1.758673	-3.226436	0.921861
H	-2.452330	-3.866928	0.338752
H	-2.100764	-3.248807	1.980830
C	2.461887	-3.008420	-0.753605
C	3.860084	-3.158146	-0.662204
H	4.392912	-3.644649	-1.496764
C	4.593337	-2.707032	0.454604
C	3.889026	-2.067530	1.494961
H	4.442041	-1.698327	2.375159
C	2.490999	-1.896869	1.456438
C	1.782960	-2.370038	0.320362
C	1.714521	-3.474441	-1.980771
H	2.418360	-3.757486	-2.787798
H	1.035099	-2.681464	-2.363597
H	1.083930	-4.366591	-1.770760
C	6.086515	-2.931893	0.547675
H	6.579082	-2.166763	1.181385
H	6.567828	-2.916905	-0.451460
H	6.312120	-3.922973	1.000314
C	1.766248	-1.209465	2.587678
H	2.451616	-0.984247	3.427705
H	0.929880	-1.825051	2.980561
H	1.314572	-0.253458	2.240845
C	-3.107038	-1.237899	0.434129
C	-3.985194	-1.443517	-0.657925
C	-5.298296	-0.946209	-0.549582
H	-5.984413	-1.086080	-1.402000
C	-5.757672	-0.278351	0.602079
C	-4.863579	-0.123161	1.679253
H	-5.202168	0.393658	2.593123
C	-3.539637	-0.595614	1.621603
C	-3.530842	-2.145559	-1.914644
H	-4.357747	-2.213686	-2.648331
H	-3.178296	-3.181080	-1.712597
H	-2.682144	-1.604400	-2.386197
C	-7.158385	0.288291	0.664127
H	-7.531278	0.351534	1.706697
H	-7.874836	-0.321146	0.076159
H	-7.184330	1.317909	0.243752
C	-2.590600	-0.354652	2.771126
H	-3.129908	0.021977	3.662311
H	-1.832081	0.407615	2.486659
H	-2.035641	-1.268958	3.070663
C	1.703934	0.227584	-0.827485
H	2.139689	-0.558006	-1.479731
C	2.654047	1.253655	-0.419178
C	4.045310	1.072412	-0.336209
H	4.467878	0.094436	-0.614426
C	4.894811	2.110060	0.123724
H	5.978798	1.929064	0.198797
C	4.375155	3.353795	0.474157
H	5.033607	4.161403	0.831299
C	2.974768	3.603470	0.359455
C	2.366341	4.854656	0.662096
H	2.982832	5.693333	1.023537
C	1.003736	5.007580	0.478590
H	0.498374	5.964044	0.674004
C	0.221015	3.911152	-0.012615
C	2.110355	2.545822	-0.088569

N -0.640247 2.242728 0.175169<br>N -2.663586 1.379361 -0.098274<br>N 3.658841 -0.887203 -0.791238<br>Cl -1.179985 -0.775089 2.681933<br>Cl -1.244327 -1.863975 -1.608389<br>Ru -0.777506 -0.772432 0.382858<br>C 5.286589 0.157596 -2.157149<br>N 5.637430 1.066629 -2.812758	N 0.348045 -2.360135 0.331763<br>N -1.797611 -1.841576 0.407888<br>N 0.754327 2.700914 -0.265744<br>Cl -0.446458 -0.339144 -2.692793<br>Cl -2.252979 1.590724 -0.363537<br>Ru -0.134800 0.509937 -0.517055<br>C -1.157458 4.256687 -0.295771<br>N -2.121769 4.905125 -0.485035
***trans*-15**: -2493.47780616 A.U.<br>Ru -0.041677 0.544124 0.001754<br>Cl -0.292873 0.639713 -2.375798<br>Cl -0.292053 0.626551 2.379581<br>N 0.557882 -2.548962 0.013908<br>N -1.591185 -2.107334 0.014755<br>C -0.350051 -1.526591 0.006412<br>C -0.053535 -3.897180 0.009067<br>H 0.299364 -4.483487 0.884323<br>H 0.249560 -4.452717 -0.905218<br>C -1.556003 -3.586902 0.055050<br>H -2.115303 -4.008271 -0.806964<br>H -2.046082 -3.955435 0.981641<br>C 1.992397 -2.488426 0.001835<br>C 2.674821 -2.495509 -1.241731<br>C 4.084405 -2.497755 -1.222517<br>H 4.625414 -2.492666 -2.183983<br>C 4.817215 -2.504087 -0.018148<br>C 4.101775 -2.500017 1.196728<br>H 4.656289 -2.496599 2.150476<br>C 2.692743 -2.497883 1.235450<br>C 1.917529 -2.451671 -2.547820<br>H 2.612868 -2.483784 -3.409386<br>H 1.302309 -1.527880 -2.628628<br>H 1.217732 -3.308806 -2.650705<br>C 6.329675 -2.550398 -0.028888<br>H 6.695711 -3.601133 -0.024640<br>H 6.761967 -2.053304 0.863270<br>H 6.748812 -2.064742 -0.933474<br>C 1.952576 -2.457702 2.551472<br>H 1.330816 -1.538991 2.638178<br>H 2.659289 -2.482648 3.403981<br>H 1.261182 -3.320461 2.664764<br>C -2.892903 -1.482758 0.004720<br>C -3.546088 -1.268067 -1.238260<br>C -4.836175 -0.702823 -1.221507<br>H -5.340833 -0.519217 -2.185307<br>C -5.499318 -0.381114 -0.022045<br>C -4.852264 -0.682697 1.190685<br>H -5.369143 -0.481376 2.144345<br>C -3.562871 -1.249462 1.234621<br>C -2.946469 -1.620153 2.562142<br>H -1.974448 -1.104972 2.713271<br>H -2.764073 -2.714875 2.639294<br>H -3.618818 -1.341017 3.397196<br>C -2.905322 -1.649852 -2.550631<br>H -2.597997 -2.718044 -2.566488<br>H -1.994522 -1.042343 -2.741423<br>H -3.610554 -1.494001 -3.390670<br>C -6.862413 0.273934 -0.038854<br>H -7.476044 -0.075498 -0.894647	***cis*-15**: -2493.47587203 A.U.<br>Ru -0.133259 0.369034 1.113161<br>Cl -0.478810 1.934984 2.885899<br>Cl -0.348703 -1.413901 2.723395<br>N 0.575668 -2.080556 -0.666696<br>N -1.596159 -1.714935 -0.672925<br>C -0.397950 -1.210747 -0.246125<br>C 0.041865 -3.220738 -1.445765<br>H 0.426481 -4.180826 -1.045966<br>H 0.371358 -3.142562 -2.506831<br>C -1.471223 -3.063443 -1.273643<br>H -2.033505 -3.113430 -2.228598<br>H -1.903344 -3.820675 -0.583748<br>C 2.006524 -1.948249 -0.645529<br>C 2.639230 -1.153165 -1.640127<br>C 4.044699 -1.133830 -1.689476<br>H 4.538051 -0.510752 -2.454510<br>C 4.833866 -1.891093 -0.798420<br>C 4.173935 -2.683194 0.160598<br>H 4.771414 -3.284746 0.866153<br>C 2.767224 -2.743775 0.251642<br>C 1.824765 -0.342037 -2.619682<br>H 2.477255 0.168106 -3.354643<br>H 1.220951 0.430111 -2.098672<br>H 1.106911 -0.974308 -3.184192<br>C 6.343443 -1.864053 -0.893243<br>H 6.695015 -2.346910 -1.831053<br>H 6.817762 -2.396400 -0.045150<br>H 6.732204 -0.823939 -0.905602<br>C 2.101734 -3.624572 1.279897<br>H 1.292556 -3.080297 1.816658<br>H 2.838502 -3.991398 2.021209<br>H 1.641284 -4.522386 0.809738<br>C -2.916704 -1.135986 -0.678674<br>C -3.281404 -0.333908 -1.791692<br>C -4.597851 0.156472 -1.864974<br>H -4.884837 0.783728 -2.726275<br>C -5.553868 -0.132379 -0.870634<br>C -5.164007 -0.957021 0.201099<br>H -5.900457 -1.208990 0.982695<br>C -3.862133 -1.486416 0.316936<br>C -3.517302 -2.409773 1.459269<br>H -2.529136 -2.168820 1.908137<br>H -3.482198 -3.470948 1.123399<br>H -4.284764 -2.350151 2.255639<br>C -2.275196 0.015976 -2.863654<br>H -1.801655 -0.885718 -3.308017<br>H -1.446072 0.633317 -2.455754<br>H -2.750777 0.585244 -3.686437<br>C -6.950492 0.444170 -0.939343<br>H -7.704609 -0.258085 -0.528888

H	-6.770560	1.378530	-0.136312	H	-7.241573	0.694382	-1.979753
H	-7.426202	0.074760	0.895146	H	-7.023073	1.380366	-0.342716
N	0.150271	2.589975	0.004095	N	0.187425	1.994865	-0.021140
C	1.827666	0.636698	0.001496	C	1.724406	0.312425	1.165123
C	3.765079	2.338136	0.000791	C	3.774916	1.755001	0.526705
C	2.406850	1.975529	0.002029	C	2.406231	1.442429	0.529324
C	1.438452	3.030887	0.003329	C	1.511370	2.358966	-0.107249
C	1.784397	4.416059	0.002961	C	1.938353	3.578710	-0.708490
C	3.176243	4.731105	0.001663	C	3.341419	3.840798	-0.705151
H	3.491581	5.786706	0.001316	H	3.716312	4.769336	-1.164629
C	4.132696	3.709529	0.000646	C	4.229191	2.945308	-0.100177
H	5.201588	3.976383	-0.000498	H	5.306015	3.177694	-0.093921
C	0.700835	5.354422	0.003533	C	0.920254	4.453378	-1.217763
H	0.912602	6.435268	0.003284	H	1.203422	5.404666	-1.694666
C	-0.607316	4.885908	0.004021	C	-0.413346	4.105937	-1.055333
H	-1.460250	5.582767	0.004136	H	-1.220748	4.776612	-1.389589
C	-0.864168	3.482897	0.004049	C	-0.766282	2.874900	-0.426851
H	2.549959	-0.201268	0.001258	H	2.339165	-0.419338	1.729400
C	-2.206459	2.878604	0.003654	C	-2.134608	2.450781	-0.125538
O	-2.379606	1.656257	0.002765	H	4.490337	1.080248	1.022309
H	-3.081439	3.583542	0.004171	O	-2.371608	1.379620	0.450027
H	4.540821	1.555879	-0.000348	H	-2.969185	3.133113	-0.436076
**TS1**$_{trans}$**-15**: -2493.43617055 A.U.				**TS1**$_{cis}$**-15**: -2493.42258256 A.U.			
C	1.335942	1.081992	0.287054	C	-1.005975	-1.172630	0.486263
C	2.016015	3.196039	1.134495	C	-1.057814	-2.788130	2.214255
H	1.834554	3.374705	2.218588	H	-0.552080	-2.743142	3.201665
H	2.054657	4.186142	0.634556	H	-1.164144	-3.861020	1.940749
C	3.265731	2.341617	0.881519	C	-2.406519	-2.041883	2.195123
H	3.881680	2.727214	0.037544	H	-3.275203	-2.725627	2.094851
H	3.925014	2.263098	1.770513	H	-2.568375	-1.426883	3.109798
C	-0.706853	3.639872	-0.741856	C	1.477034	-3.558516	0.232508
C	-1.962423	4.275906	-0.822424	C	2.841239	-3.903964	0.145191
H	-2.234381	4.797875	-1.755246	H	3.124880	-4.770531	-0.475667
C	-2.875280	4.258763	0.250511	C	3.844495	-3.189947	0.830198
C	-2.502051	3.583395	1.431325	C	3.451371	-2.106805	1.641412
H	-3.203565	3.557615	2.282295	H	4.215659	-1.550499	2.209971
C	-1.260124	2.932807	1.565763	C	2.103986	-1.714770	1.761686
C	-0.371219	2.963357	0.459097	C	1.124670	-2.432805	1.024610
C	0.237717	3.635118	-1.922076	C	0.437425	-4.364666	-0.507438
H	-0.174370	4.223830	-2.764834	H	0.912142	-5.174388	-1.095425
H	0.423104	2.599752	-2.281228	H	-0.152468	-3.726144	-1.201548
H	1.230131	4.063503	-1.665521	H	-0.287245	-4.843259	0.187018
C	-4.225254	4.932724	0.144507	C	5.303026	-3.566710	0.694884
H	-4.327250	5.502451	-0.800345	H	5.841782	-3.468093	1.659818
H	-4.394656	5.635349	0.987675	H	5.819923	-2.902303	-0.032217
H	-5.048670	4.187026	0.178866	H	5.426739	-4.607832	0.335160
C	-0.903872	2.200531	2.837249	C	1.716334	-0.554127	2.644374
H	-1.725696	2.269420	3.576199	H	2.553172	-0.267714	3.311484
H	0.008856	2.618213	3.314528	H	0.836343	-0.787363	3.279047
H	-0.701274	1.125886	2.641253	H	1.453292	0.332102	2.024706
C	3.592775	-0.064611	0.253117	C	-3.328180	-0.288945	0.592737
C	4.179223	-0.174261	-1.036371	C	-4.340699	-0.769015	-0.282231
C	5.071913	-1.237348	-1.273606	C	-5.370494	0.119853	-0.650479
H	5.512011	-1.341180	-2.279785	H	-6.154807	-0.239357	-1.337693
C	5.420093	-2.161771	-0.270662	C	-5.431458	1.440907	-0.165482
C	4.882267	-1.974345	1.017883	C	-4.435470	1.866420	0.736429
H	5.178647	-2.657587	1.831465	H	-4.475633	2.890293	1.143949
C	3.985050	-0.929020	1.312417	C	-3.382415	1.024249	1.137473
C	3.933982	0.848522	-2.122542	C	-4.324674	-2.184979	-0.804487

H	3.967011	0.377926	-3.124234
H	4.723226	1.633898	-2.101663
H	2.949804	1.341784	-2.021120
C	6.337947	-3.325434	-0.570246
H	6.896117	-3.651202	0.330860
H	7.072394	-3.075328	-1.362768
H	5.756828	-4.203146	-0.930101
C	3.499735	-0.721438	2.726740
H	3.987155	-1.436964	3.417458
H	2.400261	-0.858648	2.799738
H	3.725779	0.304209	3.090441
C	-1.296220	0.219913	-0.688678
H	-1.505068	1.308719	-0.655266
C	-2.323619	-0.615033	-1.363016
C	-2.547403	-0.409100	-2.732781
H	-1.958249	0.357714	-3.259680
C	-3.484939	-1.186983	-3.458721
H	-3.617644	-1.000890	-4.536306
C	-4.223351	-2.182019	-2.826998
H	-4.946488	-2.792631	-3.391681
C	-4.056375	-2.416413	-1.433100
C	-4.801683	-3.402783	-0.715948
H	-5.527672	-4.028908	-1.260907
C	-4.606296	-3.553206	0.644955
H	-5.148888	-4.293630	1.252082
C	-3.661744	-2.698266	1.292598
C	-3.104911	-1.620630	-0.681757
N	0.931190	2.369026	0.566448
N	2.694273	1.027586	0.536521
N	-2.945563	-1.770256	0.661344
Cl	1.146362	-0.978931	-2.356288
Cl	0.156541	-1.351809	1.919866
Ru	0.330211	-0.491747	-0.232902
C	-3.435679	-2.816495	2.771582
O	-4.031393	-3.608407	3.485308
H	-2.654702	-2.102394	3.160651

H	-5.211301	-2.380485	-1.438712
H	-4.331176	-2.930832	0.019508
H	-3.411552	-2.372639	-1.410888
C	-6.522588	2.386864	-0.612728
H	-6.855108	3.048241	0.213546
H	-7.406896	1.841717	-0.999402
H	-6.158343	3.047973	-1.429698
C	-2.335333	1.516769	2.107952
H	-2.636573	2.483324	2.556156
H	-1.366861	1.671968	1.585574
H	-2.157106	0.797414	2.934331
C	1.145661	-0.294128	-1.178716
H	1.432542	-1.336541	-1.451546
C	2.281678	0.626717	-1.354293
C	3.130255	0.303251	-2.430765
H	2.950346	-0.640117	-2.971779
C	4.155379	1.171895	-2.877350
H	4.763796	0.889886	-3.750957
C	4.383310	2.374296	-2.220523
H	5.170519	3.065141	-2.563724
C	3.611989	2.721646	-1.073360
C	3.852292	3.924453	-0.342080
H	4.633737	4.618638	-0.692648
C	3.111435	4.192655	0.793198
H	3.243899	5.100775	1.400242
C	2.148155	3.225720	1.208141
C	2.562187	1.837398	-0.604277
N	-0.258013	-2.078382	1.190479
N	-2.276305	-1.181241	1.007497
N	1.883948	2.099248	0.545109
Cl	-1.173863	-1.814241	-2.472736
Cl	-0.791783	2.347750	-1.112685
Ru	-0.671512	0.007466	-1.036385
C	1.369779	3.456704	2.467579
O	1.500517	4.439287	3.179488
H	0.636582	2.630792	2.701109

14e$^-$-15: -2493.44689582 A.U.

C	1.406668	1.058346	-0.007460
C	1.675046	3.388425	0.351415
H	1.428191	3.763064	1.371381
H	1.556188	4.236596	-0.353534
C	3.063348	2.736626	0.281242
H	3.615889	3.020756	-0.642946
H	3.706988	2.982960	1.150777
C	-1.126330	2.965947	-1.430758
C	-2.483407	3.337770	-1.521719
H	-2.883364	3.634586	-2.505920
C	-3.335500	3.349314	-0.400378
C	-2.796670	2.973063	0.847341
H	-3.444600	2.977793	1.739885
C	-1.451132	2.585172	0.993146
C	-0.625529	2.580250	-0.162214
C	-0.240409	2.974631	-2.653999
H	-0.791590	3.349144	-3.538596
H	0.142551	1.957387	-2.884609
H	0.650671	3.623748	-2.513472
C	-4.794754	3.724101	-0.531302
H	-4.981775	4.336070	-1.436420
H	-5.152608	4.292810	0.351361

TS2-15: -2493.43071561 A.U.

C	1.134338	-0.907046	0.502022
C	1.345243	-2.827055	1.907267
H	1.194150	-3.895744	1.646388
H	1.120880	-2.713801	2.992632
C	2.745067	-2.308664	1.535424
H	3.379291	-2.092783	2.420424
H	3.307579	-3.019596	0.890324
C	-1.775175	-1.608539	2.100337
C	-3.165851	-1.836765	2.041426
H	-3.807854	-1.392020	2.820848
C	-3.752620	-2.624933	1.035353
C	-2.900183	-3.231165	0.088234
H	-3.332495	-3.894575	-0.679606
C	-1.506773	-3.035915	0.097440
C	-0.954431	-2.162469	1.081400
C	-1.209255	-0.847751	3.278717
H	-1.754182	0.104099	3.444037
H	-0.137718	-0.607317	3.154201
H	-1.318726	-1.446063	4.209786
C	-5.248342	-2.837619	0.971960
H	-5.501893	-3.915162	0.885687
H	-5.684510	-2.335300	0.081473

H	-5.431479	2.815531	-0.610374	H	-5.759485	-2.435293	1.869149
C	-0.915395	2.140823	2.335435	C	-0.632725	-3.754112	-0.899300
H	-1.699631	2.200408	3.114782	H	-1.213473	-4.517355	-1.453272
H	-0.059146	2.762633	2.674362	H	0.221790	-4.265424	-0.408319
H	-0.552150	1.091452	2.294509	H	-0.205039	-3.038337	-1.636853
C	3.783135	0.327615	0.472713	C	3.624298	-0.329531	0.333975
C	4.638229	-0.043592	-0.598851	C	4.187383	0.648083	1.188711
C	5.663237	-0.976137	-0.335258	C	5.378450	1.282355	0.786029
H	6.320126	-1.282894	-1.166622	H	5.816387	2.053672	1.442073
C	5.881280	-1.511207	0.947900	C	6.016150	0.966806	-0.428593
C	5.059201	-1.062343	2.001410	C	5.435566	-0.025511	-1.244159
H	5.235505	-1.439392	3.022823	H	5.920118	-0.291725	-2.198881
C	4.016384	-0.141378	1.794818	C	4.248492	-0.692146	-0.887599
C	4.522056	0.576733	-1.971733	C	3.500727	1.059104	2.469693
H	5.063738	-0.029784	-2.724025	H	4.171536	1.669995	3.105462
H	4.976350	1.592904	-1.987499	H	3.157441	0.190184	3.069201
H	3.469103	0.665337	-2.305017	H	2.600635	1.670864	2.242903
C	6.958500	-2.544181	1.190707	C	7.275203	1.683558	-0.863364
H	7.428914	-2.419641	2.187660	H	8.033602	0.975624	-1.258389
H	7.755865	-2.494839	0.422075	H	7.734960	2.249473	-0.028494
H	6.533725	-3.571790	1.159090	H	7.057883	2.409381	-1.677371
C	3.177384	0.336026	2.956129	C	3.642851	-1.723234	-1.808371
H	3.628554	0.030469	3.920421	H	4.310376	-1.922301	-2.669369
H	2.157559	-0.099675	2.903005	H	2.659777	-1.382502	-2.202697
H	3.067242	1.440782	2.963525	H	3.459987	-2.692807	-1.296485
C	-1.061890	-0.525031	-0.398108	C	-0.313548	1.365189	1.074358
H	-1.569246	0.303997	0.128437	H	0.295361	1.385722	2.004852
C	-1.935806	-1.601567	-0.864155	C	-1.457289	2.254818	1.106839
C	-1.581070	-2.470065	-1.914750	C	-1.607630	3.285780	2.055719
H	-0.624745	-2.306443	-2.437565	H	-0.838471	3.404434	2.836055
C	-2.439738	-3.510506	-2.346166	C	-2.700361	4.184288	2.003457
H	-2.120126	-4.158714	-3.177321	H	-2.771398	4.994557	2.746252
C	-3.674952	-3.714237	-1.739523	C	-3.681798	4.054835	1.022360
H	-4.340412	-4.527121	-2.072818	H	-4.529500	4.757092	0.978679
C	-4.095531	-2.867577	-0.673640	C	-3.604000	3.004234	0.060468
C	-5.348851	-3.031272	-0.008792	C	-4.582865	2.795968	-0.955833
H	-6.026516	-3.839055	-0.331200	H	-5.446076	3.478046	-1.026339
C	-5.690306	-2.181764	1.028373	C	-4.439758	1.730048	-1.826394
H	-6.638203	-2.259210	1.582026	H	-5.168835	1.493701	-2.615998
C	-4.764751	-1.159189	1.394530	C	-3.317639	0.861941	-1.690417
C	-3.229334	-1.796565	-0.223327	C	-2.485755	2.095026	0.102549
N	0.772112	2.278826	-0.020460	N	0.469490	-1.956076	1.107800
N	2.739059	1.298652	0.265136	N	2.467864	-1.062301	0.785191
N	-3.585513	-0.973685	0.798374	N	-2.357867	1.048627	-0.769053
Cl	1.244196	-0.494672	-2.750868	Cl	1.875133	1.787787	-1.068127
Cl	1.140082	-2.001691	1.418638	Cl	0.125732	-0.847305	-2.596213
Ru	0.773009	-0.709362	-0.471923	Ru	0.107206	0.404550	-0.497312
C	-5.096460	-0.217509	2.510510	C	-3.249236	-0.356023	-2.572495
O	-6.138710	-0.245524	3.145022	O	-4.140226	-0.622027	-3.367379
H	-4.276567	0.533519	2.711580	H	-2.329674	-0.983988	-2.444662
